# Assessing Designed Model Membrane-Targeting Peptides Found as Potential Molecular Scaffolds against Cutaneous Leishmaniasis

**DOI:** 10.1021/acsomega.6c04342

**Published:** 2026-09-16

**Authors:** Viviane A. C. Santana, Danúbia B. Martins, Vanessa Albuquerque, Anna Karoline Aguiar Fleuri Balbi, José Eduardo Ferraz Souza, Andre Gustavo Tempone, Marcelo Dias-Baruffi, Thalita Riul, Dayane S. Alvares, Marcia Perez dos Santos Cabrera

**Affiliations:** † Departamento de Química e Ciências Ambientais, 135132 Universidade Estadual Paulista, São José do Rio Preto, SP 15054-000, Brasil; ‡ Departamento de Física, 135132 Universidade Estadual Paulista, São José do Rio Preto, SP 15054-000, Brasil; § Laboratório de Fisiopatologia, 196591 Instituto Butantan, São Paulo, SP 05503-900, Brasil; ∥ Faculdade de Ciências Farmacêuticas, 54534 Alimentos e Nutrição - Universidade Federal do Mato Grosso do Sul, Campo Grande, MS 79070-900, Brasil; ⊥ Departamento de Análises Clínicas Toxicológicas e Bromatológicas, Universidade de São Paulo, Ribeirão Preto, SP 14040-903, Brasil

## Abstract

Recent research has demonstrated therapeutic potential against visceral leishmaniasis, a neglected disease with high prevalence and severity in very populated low- and middle-income regions; however, effective treatments for the cutaneous or mucocutaneous forms remain limited, despite their higher incidence. The negatively charged composition of *Leishmania* membranes differentiates it from the zwitterionic nature of mammalian cells, providing a basis for selective targeting by cationic peptides. Here, designed peptides (DecP-9, DecP-17, and DecP-18), spanning a broad range of charge and hydrophobicity, were evaluated and displayed distinct antileishmanial activity and selectivity, including effects on amastigote-infected macrophages. Biophysical studies revealed their interaction profiles: (i) DecP-9, with helical structure and strong lytic activity, showed high activity but low selectivity; (ii) DecP-18, enriched in β-stranded structures, exhibited limited partitioning, low lytic activity, and membrane translocation, resulting in low antiparasitic activity and high mammalian cell survival; (iii) DecP-17 displayed intermediate behavior, reducing intracellular amastigotes without significant cytotoxicity. Although there is a scenario of valued and challenging features in the development of therapeutic peptides, it seems that to find drug peptides that will cutaneously act, but whose ultimate target is the amastigote form contained inside the macrophage, a deeper understanding of membrane processes is required.

## Graphical abstract

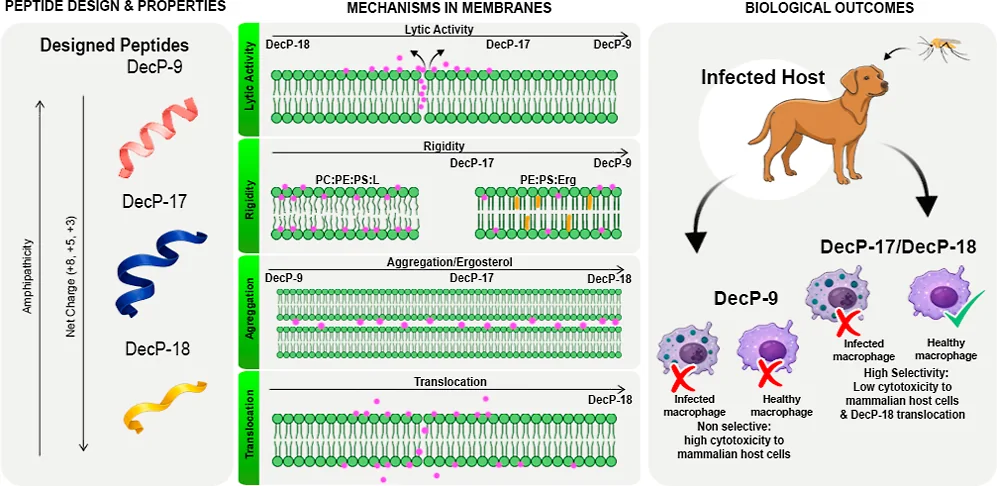

## Introduction

Peptide-based drug development shows growing demand driven by a market of million dollars.^1,2^ Most research, reflected in the number of publications in the last 10 years and in the number of clinical trials, particularly focuses on type II diabetes mellitus, cancer, Alzheimer’s disease, cardiovascular disease, and inflammation, with some other important areas having also deserved efforts.^1^ Among the features that underscores the interest in therapeutic peptides are their broad spectrum of bioactivity, low immunologic incompatibility, high selectivity, water solubility, intermediate molecular weight between small molecules and proteins, and relatively affordable production costs.^1–3^ However, peptide therapeutics still face several challenges, including enzymatic degradation, rapid elimination, limited bioavailability, and feasibility for oral delivery.^1^

In this scenario of valued and challenging features in the development of therapeutic peptides, research in the field of neglected diseases could be further expanded, profiting from advances in areas like anticancer or antimicrobial peptides that can directly kill cells and new technologies in the routes of administration. Peptides can be designed to target receptors and/or different membranes, favoring tissue penetration, with high specificity and selectivity. When peptides target phospholipid membranes, they may circumvent the emergence of drug resistance.^1,2,4^ Current treatments face limitations related to administration routes, significant side effects, and high costs, which cause poor compliance to the treatment.^2^ Leishmaniasis, one of the neglected diseases with high prevalence and severity in very populated low- or middle-income regions, represents high risk of infection and urgently requires new medicines.^2,5,6^ Depending on the etiological species, the disease is called visceral, cutaneous, or mucocutaneous and impairs in several degrees the life and health of individuals.^5,6^ Recent research shows promise for drug development for visceral leishmaniasis; however, the same cannot be said for the cutaneous or mucocutaneous forms of the disease, even though the cutaneous form has much higher incidence. In the latter forms of the disease, parasites in the persistent amastigote phase infect macrophages of the skin of the host, replicate, and spread by infecting closer cells.^2,6,7^ This mechanism of infection represents a major challenge for drug development: facing significant pH variations, leishmanicidal peptides must cross host macrophage membranes, without impairing them, to reach the acidic environment of the membrane of the resident amastigote and eliminate it.^2,3,6^

Generally acting in the micromolar range and following the steps of antimicrobial and anticancer peptides, the mechanism that leads to cellular death in the action of antileishmanial peptides involves, among others, the disruption of the membrane integrity or damage of its functions by morphological or packing alterations.^2,3,8,9^ For this, peptide molecules must be attracted to the membrane, accumulate, disturb the membrane arrangement (mainly without involving the participation of receptors), open pores or defects that enable the flux in and out of the cell at different rates concomitantly with the transference of peptide molecules to the inner side of the membrane. This translocation of peptide molecules enables the targeting of internal organelles or amastigotes inside macrophages.^7^

Some antimicrobial peptides have already shown leishmanicidal activity *in vitro* without being toxic toward mammalian cells.^4,5,10^ The composition of *Leishmania* membranes differs considerably from that of mammalian cells. The former have a predominant negatively charged character, at various degrees, depending on the form (promastigotes or amastigotes) and stage of development.^3,9,11^ In contrast, mammalian membranes are mainly zwitterionic. Their phospholipid composition has been previously discussed, and in the present work, these differences are further explored.^12^ The recent literature reports advances in therapeutic antileishmanial peptides^2^ and calls attention to the need for a desirable profile for cutaneous leishmaniasis.^6^ However, biophysical studies addressing how membrane composition influences the structural and functional behavior of antileishmanial peptides remain scarce.^2,10^ It seems that to find such drug peptides that will cutaneously act and whose ultimate target is the amastigote form contained inside the macrophage, a deeper understanding of membrane processes is required.

To this end, we started using computational methods, molecular dynamics simulations in a mimetic membrane environment, and multivariate data analysis, applied to a pool of 23 known peptides for their leishmanicidal activity to extract important structural and biophysical parameters.^13^ This approach led us to design new sequences, some of which were synthesized and evaluated for the leishmanicidal activity.^12^ In summary, starting from amidated Decoralin, with +3 charges, low hydrophobicity and showing good results against*Leishmania*major promastigotes (IC_50_ 11 μM),^14^ and considering that most peptides with interesting leishmanicidal activity have longer amino acid chains,^13^ we opted to design longer peptides (14 aa). Following the design, simulations, and multivariate data analysis, DecP-11 was synthesized and evaluated.^12^ The computational studies showed 5 descriptors to be correlated with the leishmanicidal activity: net charge, backbone solvation by the mimetic membrane environment, solvent-accessible surface area, and mean volume per residue. These parameters are related to electrostatic, hydrophobic, and steric effects.^13^

In the present work, to improve our understanding of the effects of charge and hydrophobicity on the leishmanicidal activity of peptides connected to their activity and selectivity toward membranes, a series of biophysical studies with three novel peptides were performed considering lipid bilayers that mimic those of promastigotes,^15^ infected macrophages,^16^ and mammalian cells.^17,18^ Circular dichroism, zeta potential, dye leakage, vesicles size, anisotropy of DPH, and bilayer translocation experiments evaluated, respectively, the peptides’ secondary structure changes, the electrostatic contribution to the binding, the lytic activity, the aggregation of vesicles, bilayer rigidity, and the internalization of peptide in vesicles. The three novel analog peptides were chosen from a pool designed after Decoralin^13^ and DecP-11:^12^ DecP-9, DecP-17, and DecP-18 (Table 1 and Figure 1) and compared with those previously studied, DCN (amidated Decoralin) and DecP-11.^12^

**Table 1:** Peptides Characteristics Used as a Reference and Their Analogues Designed after DCN, Their Respective Molecular Weight (MW), Net Charge (z) at pH 5.5 (Protonated Residues in Bold Letters) and Hydrophobicities <H> Calculated according to Eisenberg et al. (1984).^26^

peptide	sequences	MW^a^	z	<*H*>
**DCN** ^14^	**S**LLSLI**RK**LIT_NH2_	1255.6	+3	0.028
**DecP-11** ^12^	**S**WLSLI**RK**LINSLL_NH2_	1655.3	+3	0.014
**DecP-9**	**S**WLSLI**R**NLL**K**SLL_**NH2**_	1654.7	+3	0.000
**DecP-17**	**P**WL**H**LI**RK**LILI**H**L_**NH2**_	1763.8	+5	0.106
**DecP-18**	**RK**W**K**LILI**RK**IV**RK**_**NH2**_	1849.2	+8	–0.394

a The MW was obtained from mass spectrometry analysis by the supplier.

**Figure 1:**
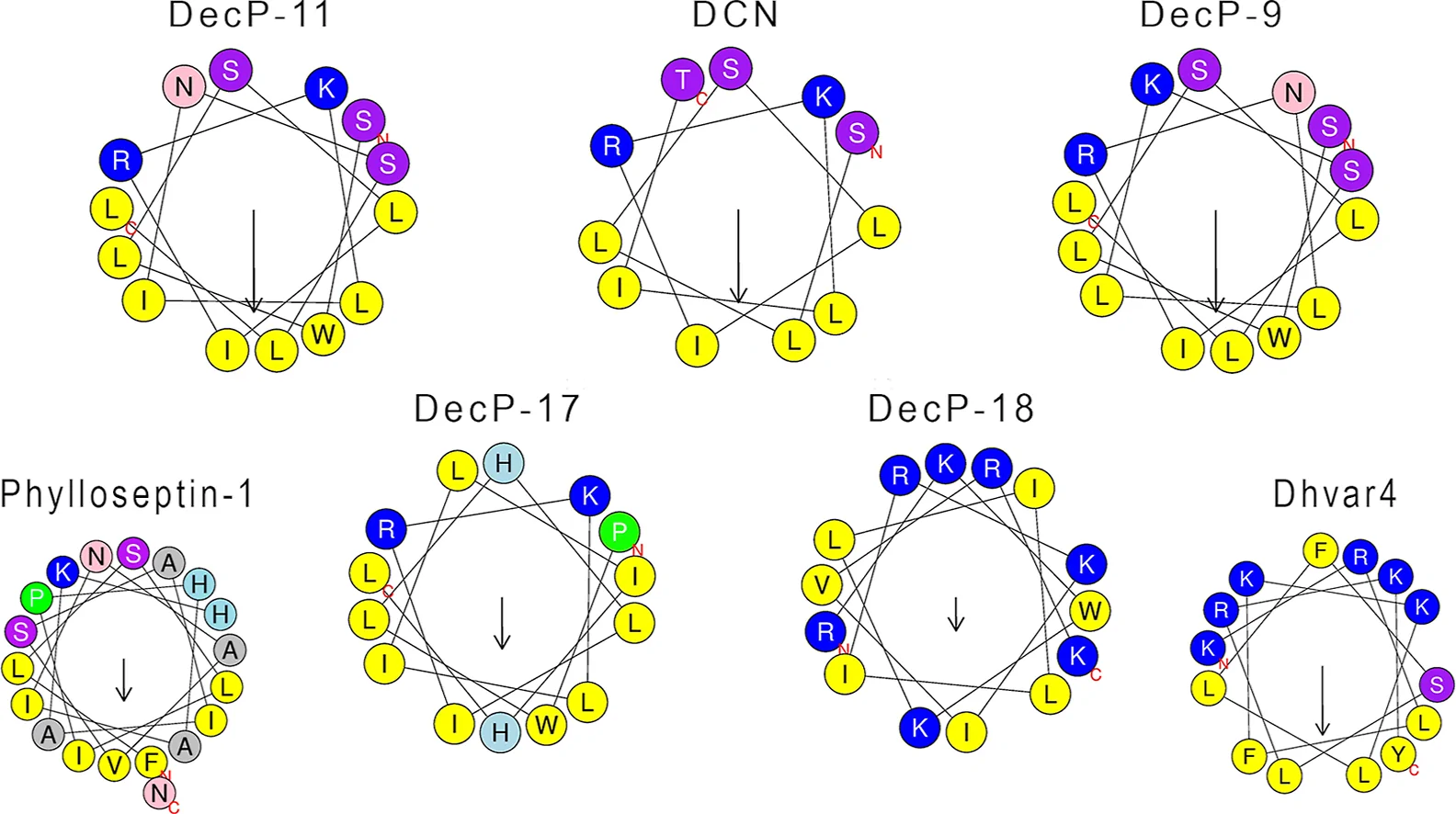
Helical wheel projections of the peptides DecP-9, DecP-17, DecP-18, in comparison with DCN, DecP-11, Phylloseptin-1, and Dhvar4, illustrating their amphipathic character quantified by the hydrophobic moment (arrows). N and C (small in red) indicate the N- and C-termini, respectively. Plots generated by HeliQuest (https://heliquest.ipmc.cnrs.fr/index.html).^27^

DecP-9, while presenting a different distribution of charged residues in relation to DecP-11, was chosen for the sequence similarities and to explore small differences in relation to backbone solvation by the mimetic membrane environment and solvent-accessible surface area.^13^ Additionally, DecP-17 and DecP-18 were designed to further explore the impact of these parameters and net charge, in line with results previously obtained,^13^ and aiming to reduce cytotoxicity. The sequence of DecP-17 was also derived from DecP-11 but mutations were inspired by Phylloseptin-1 (PH-1), a peptide from amphibian source that also has two His residues in the sequence and shows a very low IC_50_ value (0.2 μM) on *Leishmania amazonensis* promastigotes.^21^ Phylloseptin-1 shows low hemolytic activity and disruptive action on cell membranes,^22^ but also shows some toxicity at concentrations around 100 μM on mouse peritoneal macrophages,^21^ even though it reveals promising selective characteristics. Thus, DecP-17 includes two His residues, which are pH-sensitive and could adapt to the physiological external environment of the parasitophorous vacuole and become more charged in its acidic internal environment,^3,19,20^ increasing the net charge of the peptide from +3 to +5. His residues were placed at the 13th position as in PH-1 and in the fourth position to promote H-bonds with the positively charged N-terminal and the eighth cationic residue (polar face). The 11th and 12th residues were substituted to increase hydrophobicity. DecP-18 was inspired by the promising results shown by the peptide Dhvar4 on promastigotes and amastigotes of *Leishmania donovani*.^19^ Dhvar4 is a variant of the active domain of histatin 5, comprising amino acid residues 11–24, with high mean hydrophobic moment (0.439).^23^ It was shown to induce very fast leakage and to translocate on anionic phospholipid bilayer composed by phosphatidylcholine (PC) and phosphatidylserine (PS; PC/PS 9:1 molar ratio) and over the plasma membrane of its target cells.^24^ To design DecP-18 with +7 positively charged residues at physiological pH, even more mutations had to be introduced in the sequence of DecP-11. Charged residues were kept as RK, as in DecP-11. The hydrophobic W (spectroscopic probe) was maintained at the third position, the charged residue at the fourth position, and then a longer hydrophobic sequence (LILI) was kept as in DecP-17. The presence of more Arg residues in DecP-18 was intended to better withstand salt-induced shielding effects, improving electrostatic interactions with cell membranes.^2,25^

In summary, biophysical studies using model membranes and correlated with the leishmanicidal activity of these novel peptides revealed: (i) DecP-9, with the higher helical structure content, higher partitioning and stronger lytic activity, showed high efficiency but poor selectivity; (ii) DecP-18, enriched in β-stranded structures, exhibited limited partitioning, low lytic activity, and membrane translocation ability, resulted in low antiparasitic activity and high mammalian cell survival; (iii) DecP-17 displayed intermediate behavior in relation to the helical content, partitioning and lytic activity but while unable to translocate model membranes it has reduced the contents of intracellular amastigotes without significant cytotoxicity.

## Results and Discussion

### Antileishmanial Activity and Cytotoxicity of Peptides

Considering the different tissue distribution of *Leishmania spp.* parasites and their distinct membrane compositions,^2,6^ two series of experiments were carried out by independent laboratories, one with *Leishmania (L.) infantum*, representing the visceral form of the disease, and the other with *Leishmania (L.) amazonensis* and *Leishmania (V.) braziliensis*, representing the cutaneous form. The experiments considered the promastigote- and amastigote-infected macrophage forms of the parasites and evaluated the peptide's cytotoxicity in relation to peritoneal macrophages and mammalian NCTC cells to bring a wider perspective on the development of peptides with antileishmanial activities.

Previous studies with peptides DCN and DecP-11 showed that their antileishmanial activity is associated with a high cytotoxicity on NCTC cells.^12^ DecP-9, DecP-17, and DecP-18 were designed aiming to improve selectivity with a focus on the amastigote form of the parasite. As a complement of the previous work, which just considered *L. infantum*, in the present work, IC_50_ values against *L. amazonensis* and *L. braziliensis* were also determined for DCN and DecP-11 (Table 2).

**Table 2:** *In Vitro* Antileishmanial Activity; Inhibitory Concentration (IC_50_) of Peptides against *L. ( L). infantum*, *L. (L.) amazonensis*, *L. (V.) braziliensi* *s* (Promastigote and Amastigote Forms), and Cytotoxicity (CC_50_) Values in Peritoneal Macrophages, NCTC Cells, and Infected Macrophages, Including Respective Controls^a^

parasites/cells	Forms	IC_50_/CC_50_ (μM)					
		DCN	DecP-9	DecP-11	DecP-17	DecP-18	controls +
*L. ( L). infantum*	Promastigote	76^b^	16.4 ± 0.7	114^b^	NA	NA	5.1 ± 0.6^d^
SI^c^		0.9	0.9	0.1	ND	ND	39.2^d^
*L. (L.) amazonensis*		77 ± 21	41 ± 14	18 ± 2	NA	NA	0.05 ± 0.04^e^
SI^c^		0.9	0.4	0.3	ND	ND	5.76^e^
*L. (V.) braziliensis*		86 ± 11	45 ± 9	36 ± 16	NA	NA	0.05 ± 0.04^e^
SI^c^		0.8	0.4	0.2	ND	ND	5.76^e^
*L. ( L). infantum*	amastigote-infected macrophages	NS^b^	NA	NS^b^	NA	NA	6.5 ± 3.0^d^
SI^c^		ND	ND	ND	ND	ND	30.7^d^
*L. (L.) amazonensis*		NA	11 ± 2	1.4 ± 0.1	NA	NA	0.18 ± 0.07^e^
SI^c^		ND	1.5	4.2	ND	ND	1.6^e^
*L. (V.) braziliensis*		68 ± 8	26 ± 3	6 ± 3	>200	>200	0.7 ± 0.1^e^
SI^c^		0.9	0.6	0.9	ND	ND	443.1^e^
peritoneal macrophages		67.54 ± 0.02	17.00 ± 0.06	5.9 ± 0.1	>200	172.3 ± 0.1	288.7 ± 0.1^e^
NCTC		62.4^b^	15 ± 9	18^b^	>200	>200	>200^d^

a At least two independent experiments were performed in triplicate or quadruplicate and analyzed by one-way ANOVA followed by Tukeýs post-test. The 50% inhibitory concentration (IC_50_ and CC_50_) was determined by logarithm regression analysis of the data.

b As previously obtained;^12^ NA, not active (IC_50_ > 100 μM); NS, not selective for the amastigote form; ND, not determined.

c Selectivity Index (SI), ratio of CC_50_ for peritoneal macrophages to IC_50_ for parasite form. Positive controls (controls +).

d Miltefosine.

e Amphotericin B.

Against *L. infantum* promastigotes, the newly designed DecP-9 showed an IC_50_ value of 16.4 μM (control miltefosine IC_50_ = 5.1 μM), 7-fold lower than DecP-11,^12^ showing that a small change in the peptide sequence improved its efficiency; however, the cytotoxicity levels of DecP-9 and DecP-11 were found to remain essentially equivalent. Both peptides are non-selective against intracellular amastigotes, being cytotoxic to infected and non-infected peritoneal macrophages (Table 2 and Figure 2). Cytotoxicity determined against NCTC mammalian cells also shows low CC_50_ values (15 μM, Table 2). In contrast, DecP-17 and DecP-18 exhibited no significant cytotoxicity toward mammalian cells, showing CC_50_ values above or around 200 μM on non-infected peritoneal macrophages and on NCTC cells.

**Figure 2:**
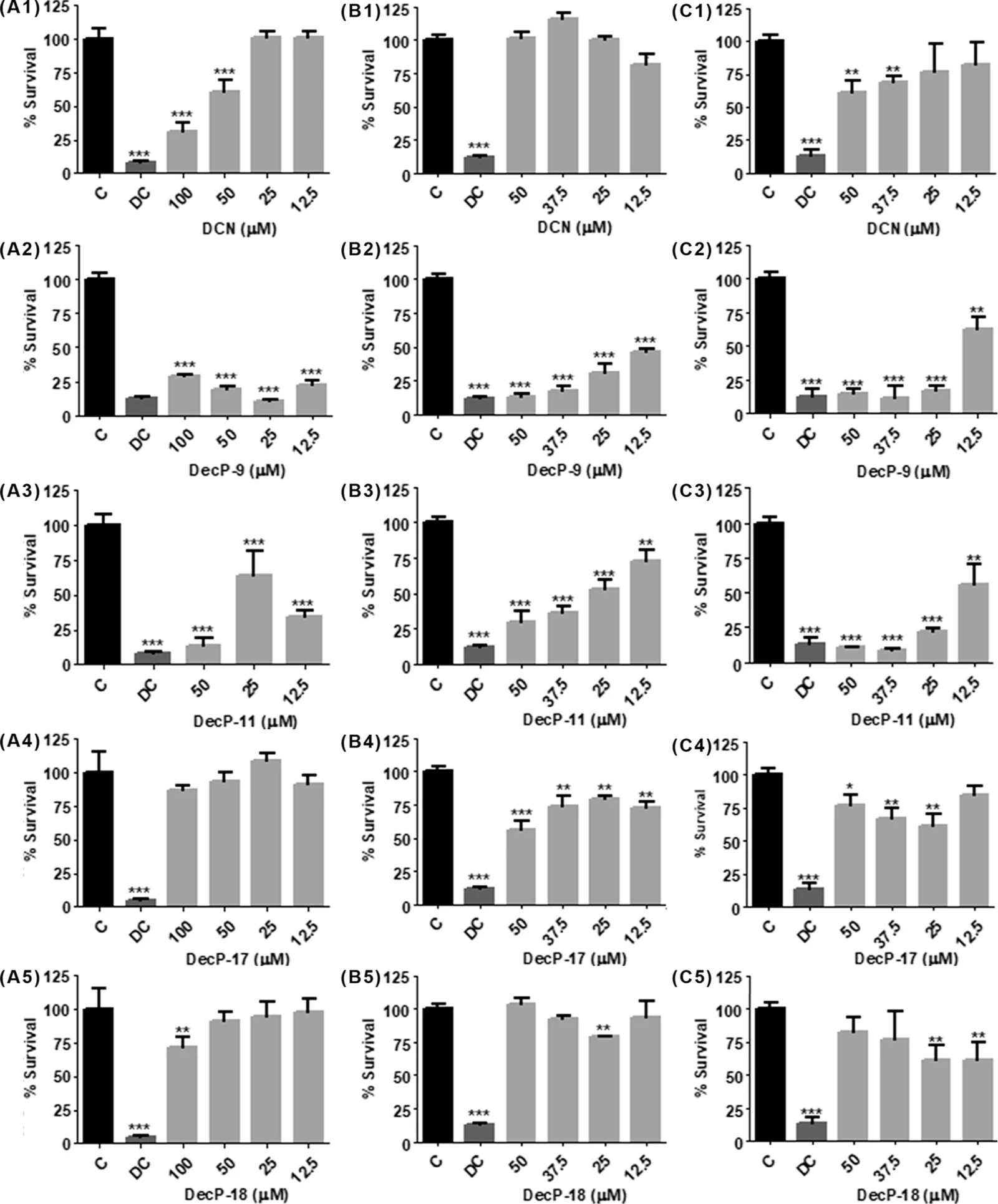
Peritoneal macrophages’ viability/survival. Susceptibility of *Leishmania*-infected and non-infected macrophages to different peptides. Columns: (A) Non-infected mice macrophages, (B) *L. amazonensis*-infected and (C) *L. braziliensis*-infected macrophages treated with peptides. Viability after 24 h was accessed by MTT assay. C, negative control without treatment; DC, death control treated with 30 μM H_2_O_2_. *, ** and *** represent *p* < 0.05, *p* < 0.01 and *p* < 0.001, respectively. Three independent experiments were performed in quadruplicate and analyzed by One-way ANOVA followed by Tukeýs post-test.

An ordinary mechanism of action among antimicrobial peptides is the permeabilization of the plasma membrane.^4^ DecP-11 was shown to display this mechanism on promastigotes of *L. infantum* and on model membranes.^12^ SYTOX Green uptake was monitored to assess membrane integrity, as this probe only fluoresces upon binding to nucleic acids within severely compromised cells; otherwise, its influx is precluded and no fluorescence can be measured. Using this approach, it was observed that the analog DecP-9 rapidly induced membrane permeabilization in *L. infantum* promastigotes; after 1h, fluorescence increased by 57% and 87% at the IC_50_ and 2× IC_50_ concentrations, respectively, relative to untreated controls (Figure SI2). This result suggested that, as for DecP-11, the permeabilization of plasma membrane is implicated in the antileishmanial activity of DecP-9. This membrane-disrupting activity aligns with the established mechanism where permeabilization triggers a lethal leakage of intracellular content. A classic example is the leishmanicidal drug amphotericin B, which similarly induces pore formation, leading to a fatal loss of the parasite’s osmotic integrity.^28^

Considering promastigotes, *L. amazonensis* showed IC_50_ values of 41 μM with DecP-9 and 18 μM with DecP-11 and in *L. braziliensis* IC_50_ values were equivalent either with DecP-11 or DecP-9 (Table 2 and Figure SI1). Despite their high sequence similarity, DecP-9 and DecP-11 exhibit differences in hydrophobicity and charge distribution, to which their distinct activity against promastigotes from different species could be attributed. This finding agrees with previous studies^13^ indicating that calculated hydrophobicity is not a significant descriptor of the biological activity, despite its association with backbone solvation, which was identified as a principal component descriptor. DecP-17 and DecP-18 showed no activity against any promastigote species. Enzymes present on the promastigote are known to reduce membrane charge, which may potentially impair the initial binding of positively charged peptides.^7^

Treatment of amastigote-infected macrophages with DecP-9 and DecP-11 resulted in low IC_50_ values for *L. amazonensis* (11 and 1.4 μM, respectively), as shown in Table 2, while DecP-17 and DecP-18 displayed activity just at higher concentrations. DecP-17 shares physicochemical similarities with DecP-11, but displays significantly increased hydrophobicity and charge, being pH-dependent due to the presence of two histidine residues. On the other hand, DecP-18 differs markedly from the others, combining high net charge with a pronounced hydrophilic character, features often associated with membrane-translocating peptides.^29^ As shown in Figure 1, DCN, DecP-9 and DecP-11 exhibit a well-defined amphipathic structure, whereas DecP-17 shows imperfect amphipathicity and DecP-18 lacks it. Efficacy observed in promastigote assays may not translate to the amastigote stage due to significant differences in their respective environments and structural integrity. These life stages exhibit unique morphological and membrane profiles that influence drug susceptibility.^9,30^ Nevertheless, DecP-9 and DecP-11 are examples for which we found out that *L. amazonensis* and *L. braziliensis* amastigotes are more peptide-sensitive than promastigotes. Although opposite activity has been reported in some cases, these findings have been attributed to differences in membrane composition and properties.^31–33^

Figure 2 compares the profiles of survival among non-infected and infected macrophages with *L. amazonensis* and *L. braziliensis*. DecP-17 and DecP-18 do not significantly affect survival of non-infected macrophages up to 50 μM. Infected macrophages showed slightly reduced viability compared to non-infected cells upon treatment with DecP-17 and DecP-18. This observation led us to evaluate the number of intracellular amastigotes after treatment with these apparently inactive peptides (Figure 3). Before treatment, an average of two amastigotes per cell were observed. DecP-17 or DecP-18 significantly reduced the mean number of amastigotes of *L. amazonensis* inside infected macrophages, but not of *L. braziliensis*-infected cells at 50 μM. Notably, IC_50_ analysis indicated that both peptides were inactive, as they did not reduce cell viability by more than 50% (Table 2) even at the highest concentration tested. However, when the cell viability was evaluated across all tested concentrations, DecP-17 reduced the viability of macrophages infected either with *L. amazonensis* and *L. braziliensis* at nearly all concentrations (Figure 2), whereas DecP-18 showed a much weaker effect.

**Figure 3:**
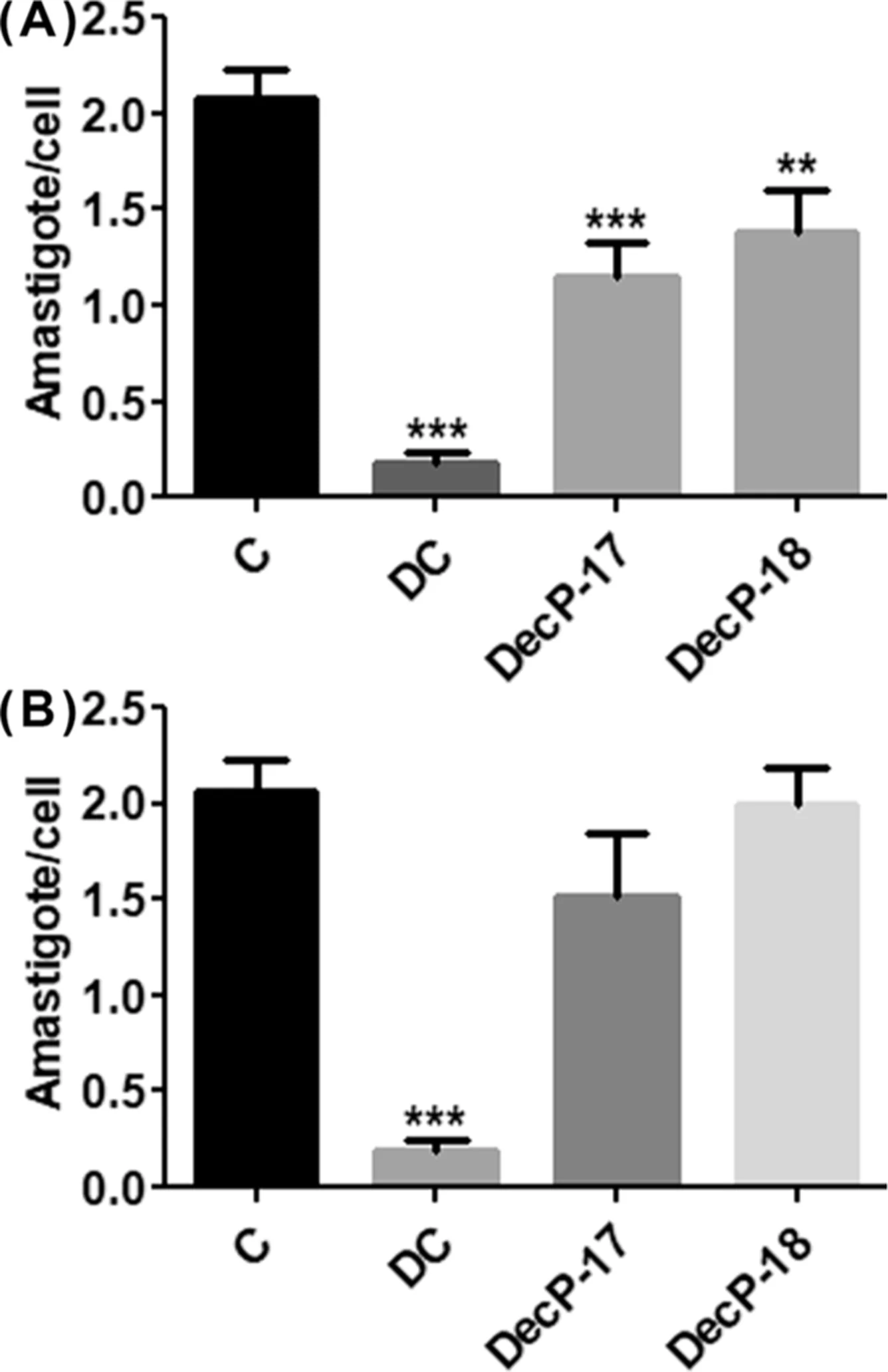
Mean number of amastigotes per infected macrophages showing peptide activity against intracellular *Leishmania* sp. amastigotes. Peritoneal macrophages were infected with (A) *L. amazonensis* and (B) *L. braziliensis* and treated with peptides DecP-17 and DecP-18 at 50 μM for 24 h. The mean number of amastigotes per infected macrophages was determined by optical microscopy. (C) negative control without treatment; DC, death control treated with 10 μM amphotericin B. ** and *** represent *p* < 0.01 and *p* < 0.001, respectively. Three independent experiments were performed in quadruplicate and analyzed by One-way ANOVA followed by Tukeýs post-test.

These findings suggest that these peptides could reach the macrophage membrane, disturb the membrane barrier function by a membranolytic mechanism or by translocation, without causing significant damage but subsequently reducing the number of intracellular amastigotes or even binding to receptors that trigger additional unknown mechanisms. Internalization of peptide molecules promoting their redistribution among the different membranes in the system may reduce their accumulation at the target membrane. DecP-17 decreased the number of internalized amastigotes of *L. amazonensis*, an effect also observed for DecP-18, although to a lesser extent (Figure 3). Compared to DecP-9, DecP-17 was designed with increased cationic and hydrophobic character, resulting in reduced targeting toward noninfected cells. Overall, these findings suggest that these sequences can be further optimized to reduce the cytotoxicity of DecP-9 and/or increase the affinity of DecP-17 for amastigote membranes.

### Assays in Mimetic Membranes

Model lipid bilayers were herein used as tools to study and compare mechanisms at the molecular level, activity, selectivity, and potency of the designed peptides, aiming to improve their physicochemical characteristics and retrofeed our design abilities. The use of mimetic composition of lipid bilayers is possible because of their resemblance to basic properties and behavior of biomembranes. However, in face of the extremely high complexity of natural membranes, some shortcomings of model membranes do not allow the direct translation of results to those obtained in cells: (i) the time scale of the peptide-membrane/peptide-cell interaction, from minutes to some hours, (ii) thickness and fluidity of membranes may not be well represented, (iii) the presence of specific outer membrane covers that may trap AMPs, (iv) the absence of other non-lipidic membrane components, and (v) the asymmetry of native membranes.^34^

#### Structural Features That Suggest Performance: Circular Dichroism (CD) Spectroscopy

CD experiments were initially performed using citrate/phosphate buffer at pH 5.5, and the secondary structure inductors, TFE solution (40%) and SDS micelles (8 mM). From the aqueous buffer to these environments, peptides underwent a conformational shift from random coil to structured patterns (Figure S3). In buffer, DecP-9 spectra exhibit predominantly α-helical characteristics, with minima near 222 and 208 nm (Figure 4A). In contrast, spectra of DecP-17 and DecP-18 are suggestive of disordered structures, as indicated by minima close to 190 nm (Figure 4B, C, respectively). Table 3 summarizes the secondary structure contributions to the spectra under different environments.

**Figure 4:**
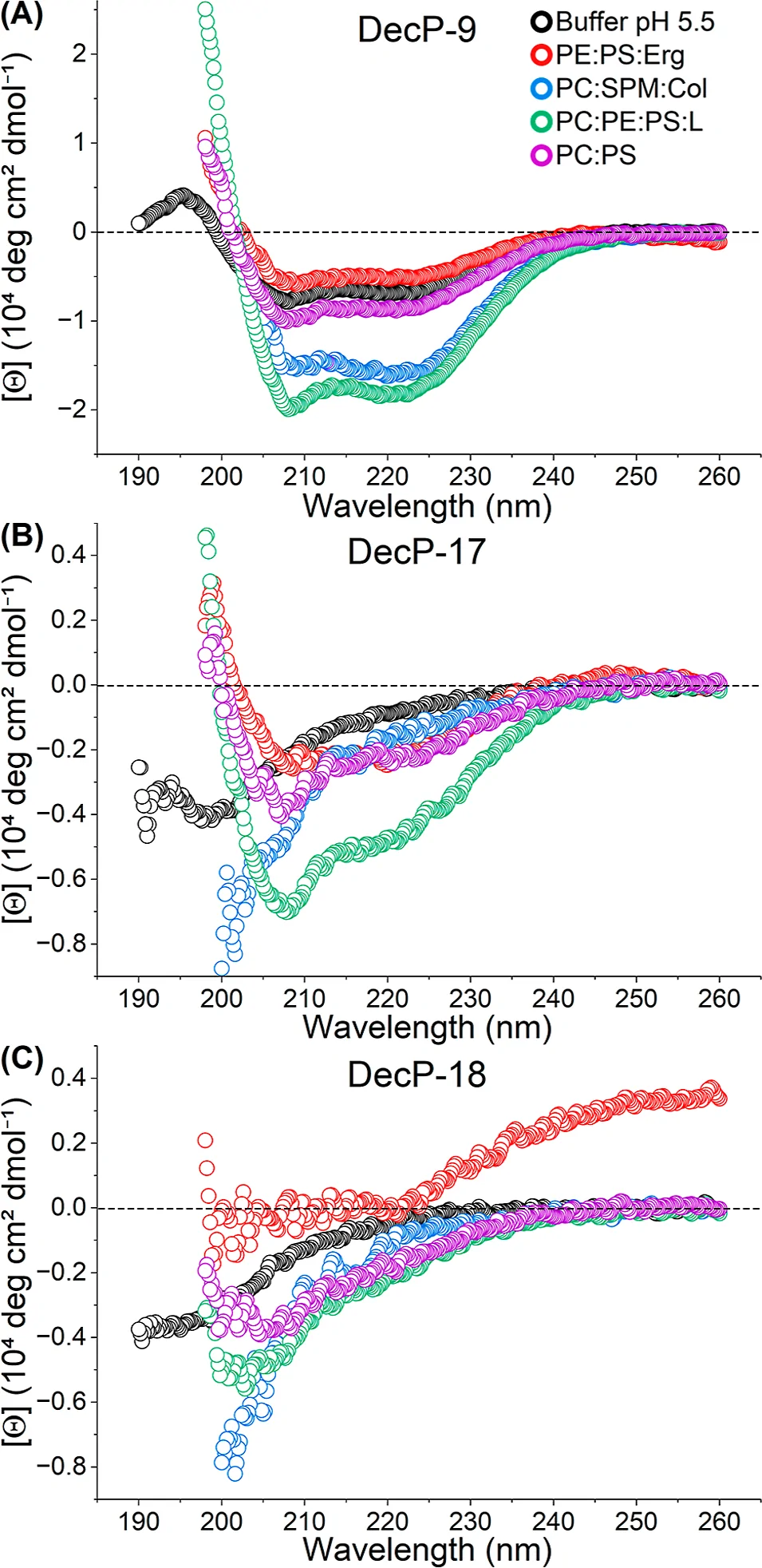
Spectra of 20 μM peptides obtained at 25 °C in buffer, pH 5.5, and in the presence of 300 μM of vesicles composed of PE/PS/Erg, PC/PE/PS/L, PC/SPM/Col, and PC/PS. (A) DecP-9; (B) DecP-17; and (C) DecP-18.

**Table 3:** Contribution of the Secondary Structures to the Spectra of DecP-9, DecP-17, and DecP-18 in Different Environments, as Calculated using Dichroweb (Birkbeck College London, London, UK).^37^

	α-helix			β-strand			turns			random		
%	D9	D17	D18	D9	D17	D18	D9	D17	D18	D9	D17	D18
Buffer	24	3	1	19	35	43	16	11	13	41	51	45
40% TFE	62	24	8	9	28	39	8	12	11	21	36	42
8 mM SDS	41	9	4	18	32	49	2	18	5	39	41	42
PC/SPM/Col^a^	45	4	2	14	29	44	13	17	15	27	50	39
PC/PE/PS/L^a^	62	16	7	11	28	47	11	14	10	16	42	36
PE/PS/Erg^a^	17	5	nd	26	42	nd	14	12	nd	43	41	nd
PC/PS^a^	41	5	4	16	42	43	15	11	7	29	41	49

a Different LUVs at 300 μM. (nd) not determined, possible intense aggregation hindering the analysis.

To assess peptide structural changes in the presence of membranes, CD experiments were conducted using membrane compositions that mimic those of the promastigote (PE/PS/Erg 47:28:25)^15^ and the amastigote-infected macrophages (PC/PE/PS/L 37:32:18:13)^16^ and comparing them to a mammalian cell membrane (PC/SPM/Chol 42:35:23)^17,18^ (Figure 4). Additionally, the electrostatic contribution was evaluated by considering the PS-rich membrane (PC/PS 71:29) as a reference.

In promastigote-mimetic vesicles, the spectra of DecP-9 showed interesting differences from those of PC/PS membrane, despite having the same anionic phospholipid content. The presence of ergosterol reduced the α-helical content of the peptide and increased its random coil structure, suggesting that membrane interaction does not favor helix formation under these conditions, likely due to increased acyl chain ordering and reduced membrane fluidity (Figure 8). In contrast, macrophage-infected mimicking membrane (PS-containing membranes lacking ergosterol) exhibited higher helical content (62%) than the PC/PS membrane (41%), despite their lower net negative charge. This result suggests a possible synergistic effect of the PE/PS mixture, similar to what has been reported for Polybia-MP1 antimicrobial peptide.^35^ Moreover, in PS-containing membranes, DecP-17 exhibited increased β-strand content driven by anionic lipid content, whereas DecP-18 displayed comparable behavior in PC/PE/PS/L (18% anionic charge) and PC/PS (30% anionic charge) systems. However, structural characterization of DecP-18 was hindered in the presence of PE/PS:Erg vesicles, even at 30% PS. Aggregation-induced light scattering distorted DecP-18’s spectra, as previously described, as well as the nonzero CD signal between 245-260 nm.^36^ In the presence of the characteristic mammalian membrane composition (PC/SPM/Chol), DecP-9 showed predominantly α-helical structures (45%), whereas DecP-17 and DecP-18 remained predominantly disordered and β-stranded.

Altogether, DecP-9 exhibits higher α-helical content in all lipid bilayers studied, while DecP-17 and DecP-18 showed higher β-strand and random coil contributions (Table 3). These features suggest that the α-helical content decreased with increasing net charge apparently independent of the hydrophobicity. DecP-17 exhibits a more pronounced difference in spectral features between vesicles mimicking macrophage-infected membranes and those mimicking mammalian membranes (Figure 4). Moreover, the structural features of DecP-9 correlate with its high cytotoxicity^12^ and low IC_50_ values observed in macrophages (Table 2), while DecP-17 and DecP-18 display features associated with low cytotoxicity and a potential ability to translocate macrophage membranes and target intracellular amastigotes (Figures 2 and 3).^24,29^ Additionally, DecP-17 and DecP-18 show higher β-strand content than DecP-9 in every tested condition (Table 3). These induced β-strands could be a consequence of the abundance of negatively charged lipids, which would allow peptides to concentrate in the membrane and favor this secondary structure.^29,36^ DecP-18 leishmanicidal activity seems to be more significant at lower concentrations (25 μM; Figure 2), suggesting that peptide aggregation could impair activity at higher concentrations. This hypothesis, correlating results in cells with structural findings in mimetic membranes, is also based on some observed features of the interaction of DecP-17 with infected-macrophage model membranes (PC/PE:PS/L), which are not observed with DecP-18, as (i) the lower β-strand content of DecP-17, indirectly points to its lower aggregation tendency, (ii) DecP-17 is slightly more helical in every membrane, and (iii) DecP-17 partitions to the amastigote-infected macrophage model membrane approximately 3-fold more than DecP-18 (Table 4).

**Table 4:** Zeta Potential Values for Pure Vesicles (ζ_0_, mV), [P]/[L] Molar Ratio at the Potential Reversal (C_rev_), and Calculated Partition Coefficients (*K*_*p*_ ± Standard Deviation) for the Different LUVs Compositions in the Presence of DecP-9, DecP-17, and DecP-18, at pH 5.5, 25 °C

	ζ_0_	DecP-9		DecP-17		DecP-18	
vesicles		K_*P*_.10^4^	C_rev_ (μM)	K_*P*_.10^4^	C_rev_ (μM)	K_*P*_.10^4^	C_rev_ (μM)
PE/PS/Erg (47:28:25)	–24.0 ± 1.2	1.8 ± 0.3	0.13	0.40 ± 0.08	0.22	0.91 ± 0.09	nd^a^
PC/PE/PS/L (37:32:18:13)	–19.9 ± 0.6	2.6 ± 0.3	0.13	1.14 ± 0.06	0.15	0.35 ± 0.04	nd
PC/PS (71:29)	–25.7 ± 1.0	1.6 ± 0.2	0.30	0.48 ± 0.01	nd	0.62 ± 0.06	nd

a (nd) Not determined, charge reversal was not observed.

#### Partitioning of Peptides Suggest Different Mechanisms of Interaction: Zeta Potential

To quantify the electrostatic contribution for the peptide-lipid interaction, zeta potential measurements were performed following the mathematical model proposed by Freire et al. (2011),^38^ which also allows the determination of the partition coefficient (K_*P*_). Figure 5A shows the adsorption isotherms of the peptides onto the outer surface of vesicles mimicking amastigote-infected macrophage membranes (PC/PE/PS/L). Other LUVs compositions are shown in Figure S4. Upon peptide addition, a progressive increase in the zeta potential is observed due to the screening of negatively charged lipids in the bilayers by cationic peptides, eventually reaching a limiting value and becoming less negative, neutral, or even positive. The ζ-potential measured at each peptide concentration was normalized to the initial zeta potential values (ζ_0_) of the anionic vesicles, i.e., in the absence of peptides (Figures 5B and S5). Normalized data as a function of peptide-to-lipid molar ratio were analyzed using eqs 3 and 4 (see Experimental Section) to determine K_*P*_ values for all peptides under different membrane compositions, considering just pre-saturated vesicles. Table 4 summarizes ζ_0_ for pure anionic vesicles along with the corresponding partition coefficients of the peptides.

**Figure 5:**
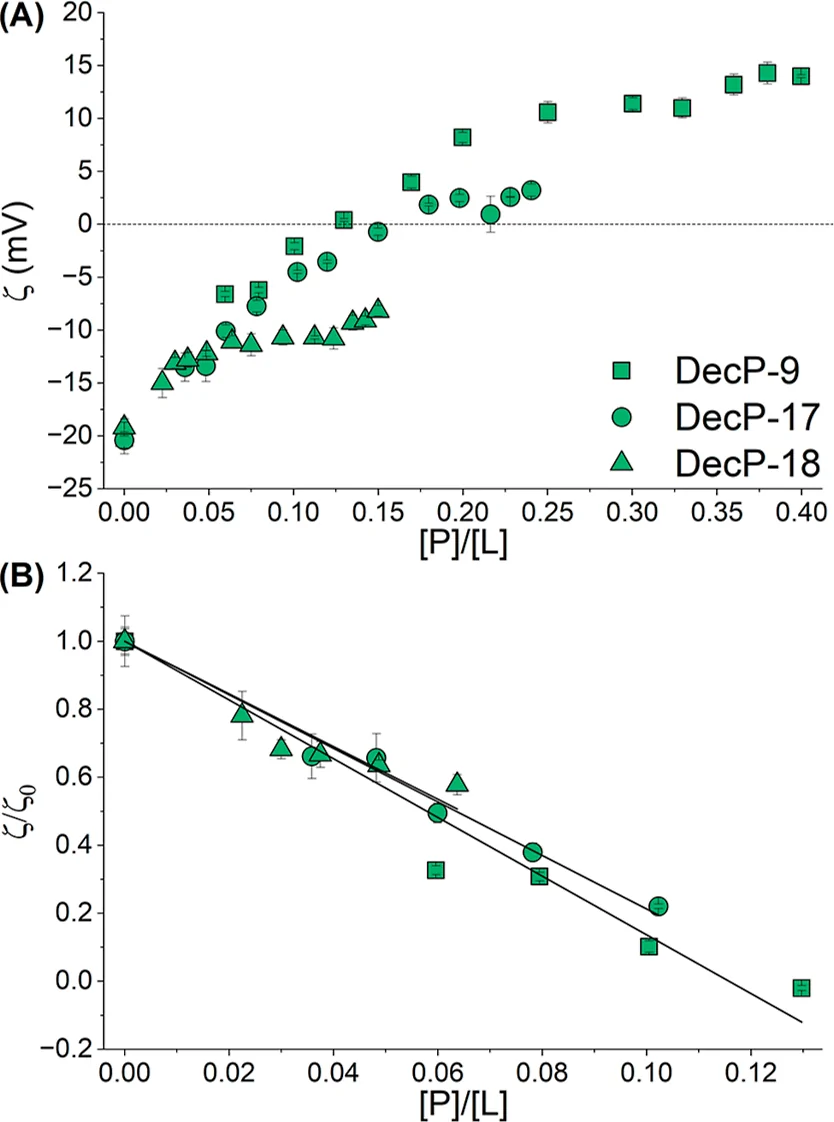
(A) Representative zeta potential isotherms obtained for indicated peptides in the presence of LUVs mimicking amastigote-infected macrophage membranes (PC/PE/PS/L). (B) Normalized zeta potential as a function of peptide/lipid molar ratio [P]/[L]. The continuous line represents the fitting according to eq 3, to determine the partition coefficients (K_P_).

DecP-9, which has the lowest cationic character, showed the most efficient charge neutralization in membranes mimicking those of promastigotes and infected macrophages. At a peptide-to-lipid molar ratio of 0.13 (Table 4), DecP-9 induced charge reversal in both cases, suggesting favorable electrostatic interaction with these membrane compositions, independent of their anionic lipid content. Interestingly, the PC/PE/PS/L system, despite its lower anionic character, exhibited a higher partition coefficient than that of PE/PS/Erg, while both membrane models showed charge reversal at the same peptide-to-lipid ratio. This indicates that the peptide partitioning and surface charge compensation are not directly proportional. In other words, a higher membrane affinity does not necessarily translate to more efficient neutralization of the interfacial charge. This behavior suggests that in PC/PE/PS/L bilayers, a larger fraction of the peptide may partition into the membrane without contributing proportionally to the ζ-potential change, possibly due to differences in insertion depth, interfacial orientation, or peptide distribution within the bilayer. Additionally, DecP-17 induced slightly higher potential reversal values, with *C*_rev_ = 0.15 in the bilayers mimicking amastigote-infected macrophages (18% anionic character) and *C*_rev_ = 0.22 in those mimicking promastigotes (28% anionic character; Table 4). Considering its net charge (+5), potential reversal would be expected at lower peptide-to-lipid ratios. In contrast, DecP-18, despite its higher net charge (+8), did not induce a charge reversal under the same conditions. These results indicate that electrostatic interactions alone do not govern peptide binding and that membrane composition and packing properties play a key role in modulating peptide adsorption and charge compensation. Furthermore, these findings suggest a distinct interaction mechanism, possibly involving peptide redistribution between both bilayer leaflets, which may indicate peptide translocation.

Comparing the isotherms obtained for PC/PS and PE/PS/Erg bilayers, which present similar anionic character (∼30%, ζ_0_ = −24 mV, Table 4 and Figure S4), DecP-9 and DecP-17 exhibited similar binding behavior with both membrane mimetics. Interestingly, higher K_*P*_ values were observed for the less negatively charged system (PC/PE/PS/L), highlighting the role of membrane composition in modulating peptide partitioning. In contrast, DecP-18 showed higher affinity for the promastigote-mimicking membranes, suggesting a possible synergistic effect of the PE/PS mixture and/or contributions from ergosterol-induced membrane ordering. The PC/PS model serves as a simplified anionic membrane model, allowing peptide interaction to be evaluated primarily as a function of membrane charge. In contrast to promastigote membrane models, this membrane lacks contributions from PE-related packing effects and sterol-induced ordering. Therefore, the presence of PE/PS and/or PE/PS/Erg alters peptide-membrane interactions, as increasing peptide charge does not enhance electrostatic affinity (Table 4).

In the zwitterionic model membrane (PC/SPM/Col), the addition of peptides leads to positive potential values, especially for DecP-9 (Figure S4), indicating stronger interaction compared to DecP-17 and DecP-18. This effect could be attributed to its lower cationicity and higher helicity, which may enhance the membrane insertion.

#### Distinct Permeabilization Profiles Revealed by Dye Leakage Assays

The permeabilization of membranes has been ascribed to many antimicrobial peptides as the main mechanism of action.^4^ These peptides target the lipid bilayer of the membranes inducing pores and/or defects that may lead to the contents leakage, membrane fusion, or even reaching other internal targets, causing cell death.^4^ The dose–response curves obtained for DecP-9 and DecP-17 (Figure 6A, B, respectively) display similar sigmoidal dependence, characteristic of cooperative processes, which have also been observed for other antimicrobial peptides. At low peptide concentrations, probe leakage is limited, while from a critical peptide-to-lipid ratio, a sharp increase in leakage is observed.^12,39^ Meanwhile, DecP-18 (Figure 6C) shows very limited and noncooperative lytic activity largely independent of the vesicle composition, particularly in PC/PE/PS/L, macrophage-infected mimicking membranes. This behavior suggests a distinct interaction mechanism, possibly involving membrane insertion or translocation without a significant disruption of membrane integrity. DecP-9 and DecP-17 permeate the three studied vesicles to a significant extent, reaching 100% leakage at low concentration within 15 min (Figure 6A, B, respectively). These peptides show intense lytic activity in the anionic and zwitterionic vesicles, however, they strongly differentiate in their biological activity toward promastigote and amastigote cells, as well as in their cytotoxicity on NCTC cells (Table 2). While DecP-9 displays high activity in the tested cells, DecP-17 exhibits very low activity against infected and noninfected amastigotes, along with low cytotoxicity. These observations, together with the low K_P_ values of DecP-17 in anionic vesicles (Table 4), lead us to hypothesize that they act by different mechanisms in which reduced partitioning to the outer leaflet could be associated with the translocation across the bilayer or aggregation of peptide molecules.

**Figure 6:**
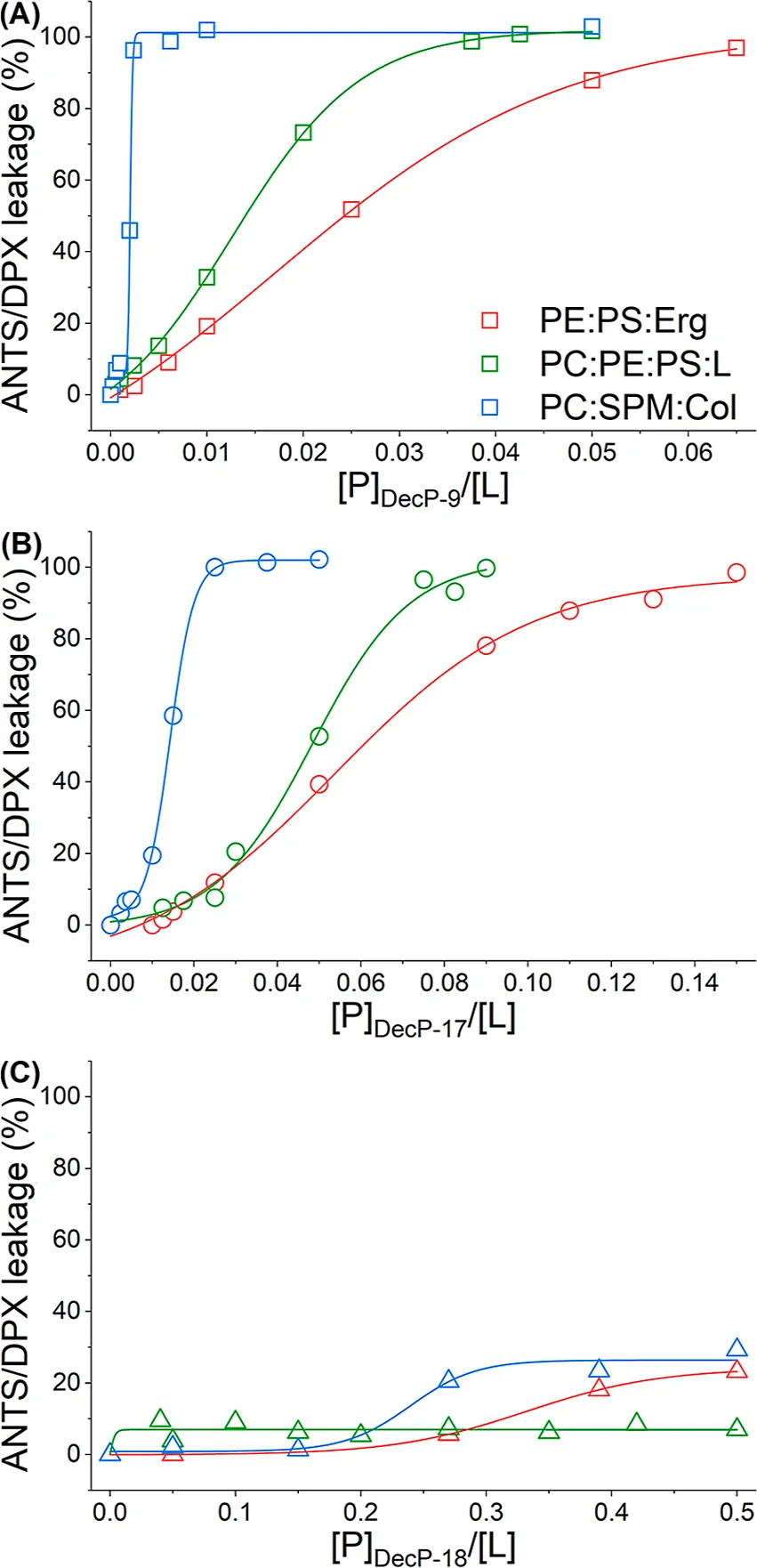
Lytic activity of peptides on 100 μM LUVs containing the probe ANTS/DPX, at 25 °C. Dose–response curves after 15 min of addition of the vesicles composed of PE/PS/Erg (red), PC/PE/PS/L (green), or PC/SPM/Col (blue). The continuous lines represent Boltzmann’s sigmoidal adjustment. (A) DecP-9, (B) DecP-17, and (C) DecP-18. Correlation coefficients ranging from 0.9775 to 0.9999.

Contrastingly, DecP-18 (Figure 6C) induces at most 20% leakage in the same vesicles, even at high concentrations (>20 μM), indicating limited destabilization. This behavior may be associated with transient formation of peptide-phospholipid defects and/or pores and suggests that translocation could occur without extensive membrane disruption.^8^ Nevertheless, negligible leakage of vesicle entrapped probes at high peptide-to-lipid ratios was also observed with penetratin and analogs that are known for assuming β-sheet structures and inducing vesicle aggregation,^36^ although penetratin was found to not translocate.^40^

#### Charge Effects on Vesicles’ Aggregation: Size Measurements–DLS

Figure 7 shows vesicles’ size in the presence of increasing peptide concentrations. None of the peptides induced significant size changes in zwitterionic and weakly anionic vesicles (PC/SPM/Col and PC/PE/PS/L, respectively). In PC/PS vesicles (∼30% anionic character) DecP-17 and DecP-18 (Figure 7B, C) also did not induce significant size changes, while DecP-9 (Figure 7A) causes a modest increase in vesicle size (up to ∼300 nm). Nevertheless, the three peptides induce significant increase in vesicle size in PE/PS/Erg, which also display an ∼30% anionic character. Taking these results together with previous results obtained for DCN and DecP-11 peptides,^12^ they suggest that the pronounced aggregation observed correlates with a synergistic effect involving PE, PS, and ergosterol. In this context, PE may favor membrane curvature and deformation, PS promotes peptide binding, and ergosterol modulates membrane ordering, collectively inducing peptide mediated inter-vesicle interaction and aggregation. PE, a lipid with negative curvature, would favor the aggregation. Despite their β-strand profiles observed by CD, DecP-17, and DecP-18 (Figure 4 and Table 3) did not induce significant size change in most membrane compositions. This indicates that β-strand alone is not sufficient to drive membrane aggregation or disruption. When considered alongside the partition coefficients (K_p_) and zeta potential data (Table 4), these results suggest that DecP-17, and especially DecP-18, interact with membranes through mechanisms that do not primarily involve surface accumulation or large-scale membrane destabilization, possibly involving peptide in an intra-vesicle redistribution of peptide molecules as previously proposed for dHP3-84 peptide.^41^ An increase in peptide charge does not necessarily enhance surface accumulation, as electrostatic repulsion between adjacent peptide molecules at the membrane interface may limit their packing density. This effect may reduce the efficiency of surface charge neutralization and favor alternative interaction mechanisms, such as peptide insertion or translocation across the bilayer.

**Figure 7:**
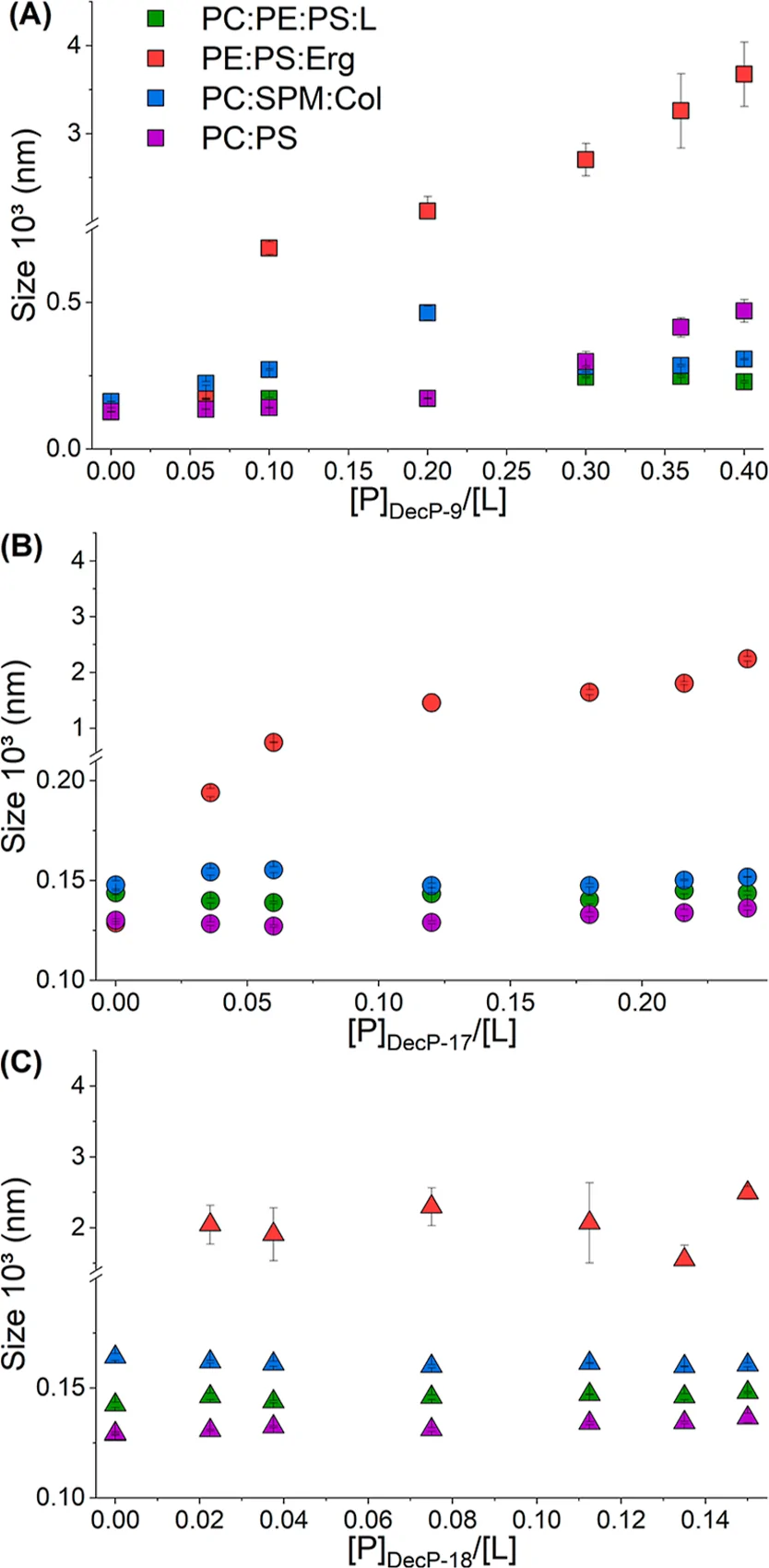
Size changes induced by peptides on indicated LUVs compositions, at 25 °C. (A) DecP-9, (B) DecP-17, and (C) DecP-18.

#### Membrane Order Assessed by DPH Anisotropy

DPH anisotropy measurements (*r*) further supported distinct modes of membrane interaction among the peptides. Positive changes of anisotropy appear as a consequence of the decrease in the probe mobility, reflecting the more dense packing of the phospholipids tail region in the bilayer. Lipid composition of the bilayer determines its rigidity.^42^ DPH anisotropy was measured as a function of peptide concentration in PC/PS, PC/PE/PS, PE/PS, and PE/PS/Erg vesicles (Figure 8). Distinct behaviors were observed for the three peptides. For peptides DecP-9 and DecP-17 (Figure 8A, B), a concentration-dependent increase in anisotropy was observed in PC/PS vesicles, indicating a progressive reduction in acyl chain mobility in this membrane composition. In contrast, in PE-containing membranes (PC/PE/PS and PE/PS), anisotropy remained nearly constant at lower peptide concentrations and increased only slightly at higher concentrations, suggesting a weaker effect on the hydrophobic core. In PE/PS/Erg vesicles, peptide DecP-9 displayed a biphasic behavior, with an initial decrease in anisotropy followed by an increase, whereas DecP-17 induced a modest increase that tended to plateau. These results indicate that PE and ergosterol modulate the way peptide binding affects bilayer organization. While PE-containing membranes appear to modulate peptide-induced perturbations at low concentrations, the presence of ergosterol alters this response more markedly, leading either to a two-step reorganization process, as observed for DecP-9, or to a limited ordering effect, as observed for DecP-17. In contrast, DecP-18 (Figure 8C) did not significantly alter anisotropy in any of the membrane compositions, indicating minimal perturbation of acyl chain dynamics. This behavior may be related to the β-stranded structure tendency that makes the peptide remain closer to the polar lipid headgroup (Table 3).

**Figure 8:**
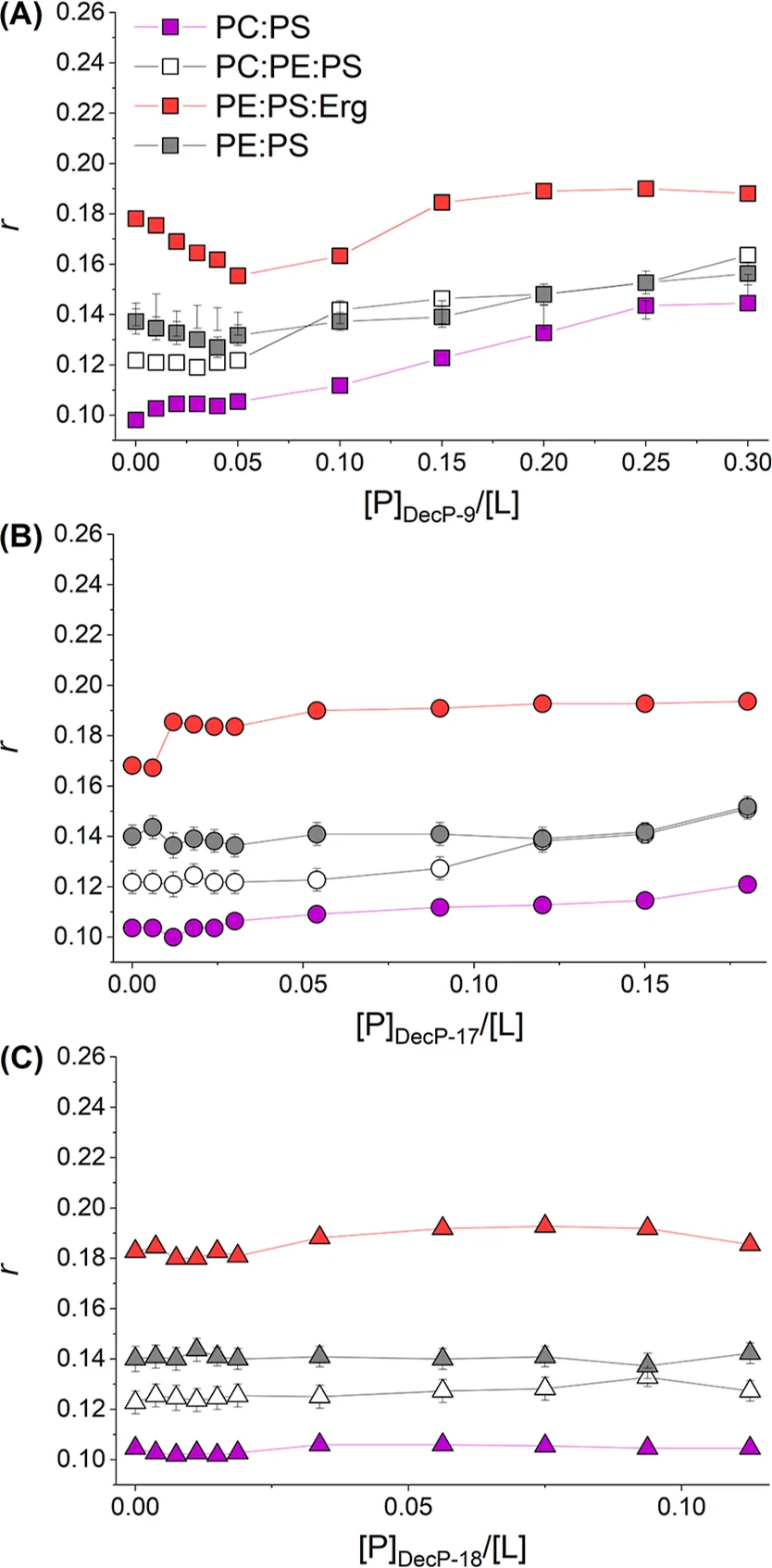
DPH anisotropy (*r*) in different LUVs measured at 25 °C as a function of peptide-to-lipid molar ratio. (A) DecP-9; (B) DecP-17; and (C) DecP-18.

#### Distinct Lytic and Translocation Mechanisms

The ability of peptides to overcome lipid membranes in a receptor-independent way, enabling their targeting to other intracellular membranes and cytoplasmic processes,^40,43^ is an interesting characteristic for peptides that are expected to reach the amastigotes inside the parasitophorous vacuoles.

The reversibility of peptide-membrane binding was investigated through two complementary experiments, referred to as the release and the dilution stage. From these assays, the fraction (υ) of DecP-17 and DecP-18 partitioned between the two leaflets of the macrophage-infected membrane model (PC/PE/PS/L) was determined (Figure 9). Translocation of the peptides was inferred when similar partitioning was observed in both stages, indicating that, within the experimental time scale, peptides were able to equilibrate across the bilayers. In contrast, peptides that do not translocate remain predominantly associated with the outer leaflet or irreversibly entrapped in the vesicles co-prepared with peptides, showing no redistribution upon dilution.^44,45^ In this sense, our results indicate that DecP-17 (Figure 9A) is unable to translocate across bilayers mimicking macrophage-infected membranes, whereas DecP-18 (Figure 9B) appears to do so. When evaluating the leishmanicidal activity of these peptides against the infected macrophages (Figure 3), the opposite behavior could be expected. However, based on their sequence composition, peptides enriched in residues such as Trp, Pro, His, or Arg have been reported to translocate across cell membranes and attack internal cellular targets.^46^ Our findings for DecP-18 in the interaction with model membranes suggest that this peptide did not remain exclusively associated with the membrane of infected macrophages, but would be somehow able to reach intracellular amastigotes, as previously observed for Dhvar4.^24^ Even though the translocation of DecP-17 (Figure 9A) across model membranes was not verified, its activity on infected macrophages (Figure 3) suggests the possibility of intracellular access, which by hypothesis could happen through membrane permeabilization. Moreover, DecP-18 emerges as an interesting, optimizable scaffold, combining leishmanicidal activity with the ability to act as a carrier for antileishmanial drugs, as proposed for other membrane-active peptides.^47^

**Figure 9:**
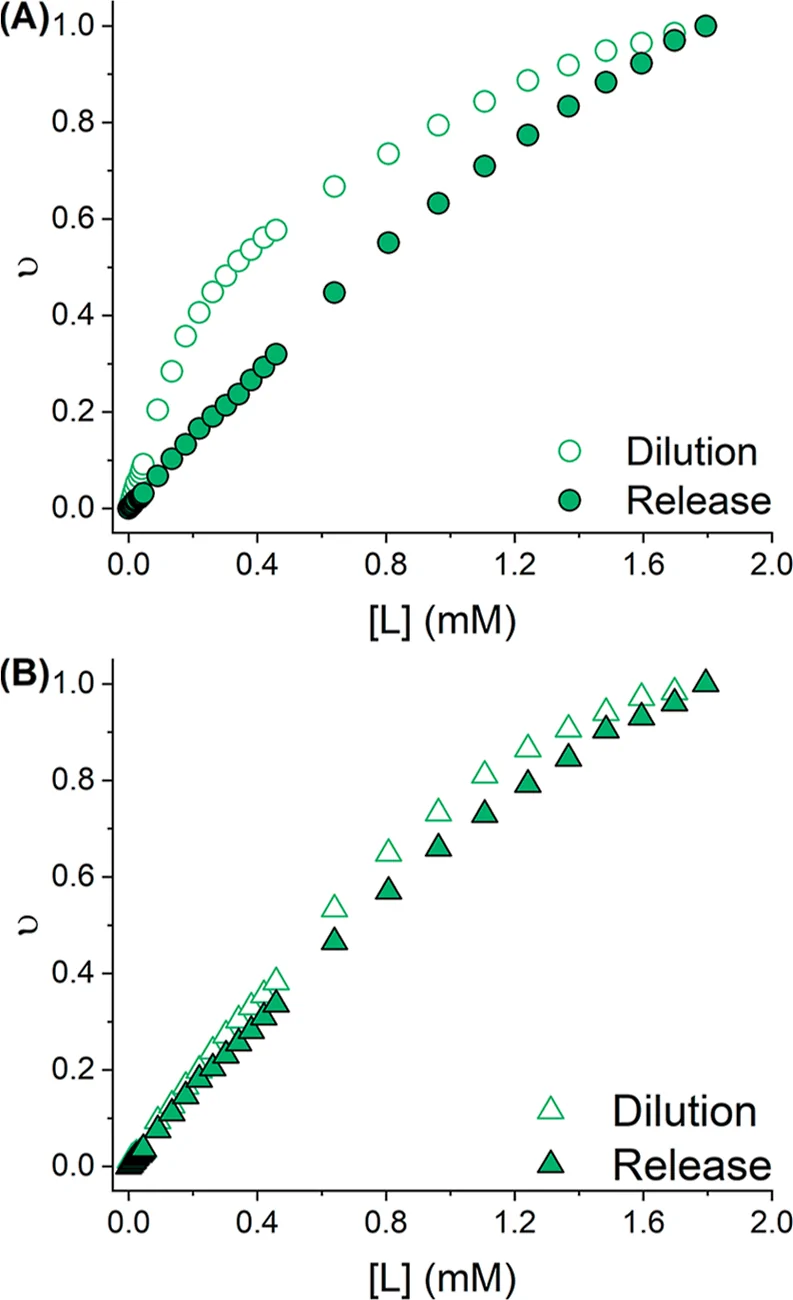
Comparison of the release and dilution stages of the translocation experiment. Fraction of peptide bound to the membrane (υ) as a function of PC/PE:PS/L LUVs concentration in citrate/phosphate buffer, pH 5.5, at 25 ^o^C. (A) DecP-17; (B) DecP-18.

Studies describing peptides’ direct translocation mechanism indicate that the process starts with peptides accumulating at the membrane surface, similar to the carpet mechanism, which induces folding and/or oligomerization.^29^ DecP-17 and DecP-18 preferentially assumed β-stranded configurations, which for short-chain peptides is indicative of oligomerization mediated by LUVs phospholipids. As a consequence the disturbed bilayer loses its barrier properties and vesicles leak, through pores or defects as preferentially occurred with DecP-17, or allows the electrostatic equilibration of peptide molecules between both leaflets of the bilayer, as indicated by the translocation of DecP-18 (Figure 9B).

## Conclusions

Several peptides, whose antimicrobial or tumoricidal activity was established, became objects of search for their possible antiparasitic profiles. The facts that bioactive peptides show a preferential interaction with membranes and that this mechanism of action reduces the chance of resistance development are certainly part of their ever-increasing research interest. In relation to the leishmanicidal activity, these observations are particularly interesting because the present forms of treatment are long-lasting, cause adverse side effects, have limited efficiency, and urgently call for the development of alternatives. Also, evidence is accumulating that suggests persistent forms of leishmaniasis.^6^

Specifically against *Leishmania*, the partitioning of peptides to the macrophage-infected membranes and their poration precede the translocation and are determinants of peptide efficiency and selectivity, this means, peptides must reach and interact with the amastigote membrane to elicit their action. In this sense, the three peptides under study exhibit rather different behavior in their interaction with lipid bilayers, which if we take as reflecting their action toward macrophage-infected membranes and amastigotes, would suggest further structural changes to improve activity for each of these peptides.

DecP-9 shows very intense leishmanicidal activity; however, it is devoid of selectivity, as verified by its high cytotoxicity toward peritoneal macrophages and NCTC cells. From the interaction with lipid bilayers, DecP-9 exhibits the highest helical structure and lytic activity in every membrane mimetic system tested, meaning a nonselective behavior. Its highest partitioning to the bilayers dependent on the anionic content shows the importance of electrostatic and non-electrostatic contributions to its mechanism of action. DecP-9, DCN, and DecP-11 have the same net charge and slight differences in their hydrophobicity, however, just DecP-11 discriminates between *L. infantum* promastigotes and NCTC cells (Table 2). This behavior suggests the importance of the relative positioning of the charged residues, Arg and Lys (Figure 1), and that new analogs of DecP-11 could be designed to increase its efficiency by working on different positionings of the charged residues within the polar face. Also, comparing the structure/activity relationships of DecP-9/DecP-11with those of DecP-17, they suggest that increasing the net charge of DecP-11 as in DecP-17 an increased antileishmanial activity parallel to a reduced cytotoxicity could be obtained.

DecP-17 and DecP-18 have increased net charges, respectively, +5 and +8 (at pH 5.5) and opposite hydrophobicities, either high for DecP-17 (0.106) or low for DecP-18 (−0.394). These peptides exhibit a lower tendency to helical structures, actually DecP-18 shows mainly stranded structures (Table 3). They have equivalent partitioning but rather different lytic activity. While DecP-17 exhibits lytic activity in the LUVs tested, DecP-18 shows no significant lytic activity but is able to translocate across membranes mimicking amastigote-infected macrophages, while DecP-17 is not. Although neither peptide showed leishmanicidal activity in the MTT assay, further evaluation for the susceptibility of *Leishmania*-infected and noninfected macrophages revealed measured activity against *L. amazonensis* and *L. braziliensis* for both DecP-17 and DecP-18 (Figures 2 and 3). Curiously, activity of DecP-18 was found at the lower concentrations tested. This could mean that the tendency to stranded structures reflects an eventual oligomerization of DecP-18 at higher concentrations that reduces its activity. An attempt to increase the leishmanicidal activity of DecP-18 while preserving its translocation ability could be carried out by repositioning its charged residues, organizing its apolar face (Figure 1).

Exploring the interplay among structural, physicochemical, biophysical, and biological profiles of leishmanicidal peptides represents a means of designing and predicting new sequences by feeding this huge volume of data to computational algorithms.

## Experimental Section

Peptides were supplied by GenScript (Piscataway, NJ) at a minimum purity of 95.7%. Stock solutions were prepared in ultrapure water, rendering clear solutions. All other compounds are >95% by HPLC analysis unless otherwise stated. All of the materials were used as supplied.

### Bioassays

#### Ethics

Animal procedures were performed with the approval of the Research Ethics Commission (CEUA IAL 02/2011) and the Animal Ethics Committee from Faculty of Pharmaceutical Sciences of Ribeirão Preto, University of Sao Paulo number 16.5.219.60.0 in agreement with the Guide for the Care and Use of Laboratory Animals from the National Academy of Sciences.

### Parasites and Mammalian Cells Maintenance

*Leishmania (L.) infantum* (MHOM/BR/1972/LD) was maintained in golden hamsters (*Mesocricetus auratus*) up to approximately 60–70 days post infection. Amastigotes were obtained from the spleen of previously infected hamsters and purified by differential centrifugation. Promastigotes were maintained in M-199 medium supplemented with 10% fetal calf serum (FBS) and 0.25% hemin at 24 °C.

Macrophages were collected from the peritoneal cavity of BALB/c mice by washing with RPMI-1640 medium supplemented with 10% FBS. The murine conjunctive cells (NCTC clone 929, ATCC) were maintained in RPMI-1640 supplemented with 10% FBS at 37 °C in a humidified atmosphere containing 5% CO_2_. BALB/c mice and Golden hamsters were maintained as described elsewhere (Reimão et al., 2012).

*Leishmania (L.) braziliensis* strain M29030 (MHOM/BR/1975/M2903) and *L. (L.) amazonensis* strain PH8 (IFLA/BR/67/PH8) promastigote forms of the parasites were cultured in Schneider’s medium (Sigma-Co, St. Louis, MO, USA) containing 20% inactivated fetal bovine serum (GIBCO), 1% l-glutamine, 10 IU penicillin, and 100 μg/mL streptomycin (USB Corporation Cleveland, OH USA) and 2% sterile male human urine in culture bottles (Corning Incorporated, Corning NY-USA) at 26 °C. For the culture of infected and non-infected macrophages, RPMI 1640 medium (Sigma-Aldrich, USA) supplemented with 10% inactivated fetal bovine serum (Gibco), 10 IU/ml penicillin, 100 μg/mL streptomycin, 2 mM l-glutamine and Hank’s balanced salts (Sigma-Aldrich) were used. Murine peritoneal macrophages were obtained from 8 weeks old Balb/c mice from Central Animal Facility of University of São Paulo – campus Ribeirão Preto.

#### Peptide Activity against Leishmania Parasites and Mammalian Cells

Stock solutions of the peptides DecP-9, DecP-17, and DecP-18 (also DCN and DecP-11, when indicated) were prepared in sterile water.

#### Determination of the 50% Inhibitory Concentration (IC_50_)

Promastigotes of *Leishmania (L.) infantum* were seeded at 1×10^6^ parasite/well on 96-wells microplate and incubated with peptides serially diluted (two-fold) in M-199 medium supplemented with 10% FBS for 48 h, 24 °C. Promastigote viability was determined using colorimetric MTT (3-(4,5-Dimethylthiazol-2-yl) −2,5-Diphenyltetrazolium Bromide) assay.^48^

Amastigotes of *Leishmania (L.) infantum* were used to infect 1×10^5^ peritoneal macrophages/well at the ratio of 1:10 (macrophage/amastigotes) for 24 h, 37 °C, 5% CO_2_. Infected macrophages were incubated with the peptides for 120 h at the same controlled conditions, in duplicate. Finally, controls as well treated macrophages, were fixed in methanol, stained with Giemsa and counted under light microscope. Miltefosine was used as a standard drug.

Log-phase promastigotes of *(L.) amazonensis* and Leishmania (V.) braziliensis (1×10^6^ cells/well) were aliquoted in Schneider medium (500 μL/well) into 96-well plates containing each peptide diluted in the same medium (500 μL/well), and the final peptides concentrations in each well ranged from 100 to 12,5 μM. Amphotericin B (ANFB) (10 μM) was used as the control drug. The plates were incubated for 24 h at 27 °C. After treatment, 10 μL of a 5 mg/mL MTT solution was added to the wells, and incubated for an additional 4 h. The formed formazan crystals were diluted after discarding the supernatant with 100 μL DMSO for OD reading at 570 nm in a Spectramax Plus microplate reader (Molecular Devices). Life control wells (C), incubated with medium alone, were defined as 100% viability, and the viability of the treated samples was expressed relative to this control. Experiments performed in quadruplicate and analyzed by One-way ANOVA followed by Tukeýs post-test. The 50% inhibitory concentration (IC_50_) was determined by logarithm regression analysis of the data.

In macrophages infected with *Leishmania (L.) amazonensis* and *Leishmania (V.) braziliensis* the activity of peptides was determined (higher concentration of 100 μM) in RPMI medium, supplemented with 10% FBS. 1 × 10^5^ peritoneal macrophages were added per well in a 96-well plate and allowed to adhere overnight. After this period, the infection with *L. amazonensis* or *L. braziliensis* was carried out in the proportion of 5 parasites to 1 cell and after 3 h peptides were added and incubated for 24 h. Amphotericin B was used as standard drug or death control. The activity of the peptides against the infected macrophages was also evaluated by MTT assay. The experiments performed in quadruplicate and analyzed by One-way ANOVA followed by Tukey’s post-test, IC_50_ values were determined by logarithm regression analysis.

#### Cytotoxicity against Mammalian Cells – 50% Cytotoxic Concentration (CC_50_). Determination on NCTC Cells

NCTC clone 929 cells were seeded at 6 × 10^4^ cells/well on 96-wells microplate and incubated with peptides serially diluted (two-fold) in RPMI-1640 supplemented with 10% FBS at maximum concentration of 200 μM for 72 h at 37 °C, 5% CO_2_. Cell viability was determined using colorimetric MTT assay in triplicate.^49^

#### Determination on Peritoneal Mice Macrophages

Peritoneal mice macrophages were incubated in 96-well plates at 37 °C and 5% CO_2_ in complete RPMI 1640 media containing 10% of fetal bovine serum. After 24 h, and after discarding the supernatant, macrophages were treated with different concentrations of the peptides (100 to 12.5 μM) in 100 μL of RPMI medium for 24 h at 37 °C and 5% CO_2_. Life control (C) was incubated only with culture medium, and death control (DC) with 30 μM hydrogen peroxide. After all treatments, the activity of the peptides against the macrophages was also evaluated by MTT assay. These assays were performed in quadruplicate.

#### Statistical Analysis

IC_50_ and CC_50_ values were calculated from sigmoidal dose–response curves. All analyses were performed using GraphPad Prism 5.0 software. Differences between groups were evaluated using t-tests or one-way analysis of variance, with *p*-values <0.05, considered statistically significant. Experiments were conducted at least with two biological replicates, each measured in triplicate or quadruplicate (technical replicates), and all assays were independently repeated at least twice to ensure reproducibility.

#### Permeabilization of the Plasma Membrane

Promastigotes of *Leishmania (L.) infantum* at the late growth phase were seeded at 2 × 10^6^ parasites/well in Hank’s medium supplemented with 10 mM glucose and incubated with 1 μM Sytox Green for 5 min in the dark. Peptides were added at the IC_50_ value and the fluorescence intensity measured during 1 h incubation. Maximum and minimum permeabilization was obtained with 0.1% Triton X-100 solution and untreated promastigotes, respectively. Fluorescence intensity was expressed as the fluorescence increase relative to basal fluorescence measured prior to peptide stimuli. Fluorescence measurements were performed at 485 nm excitation and 520 nm emission wavelengths using the FilterMax F5Multi-Mode Microplate Reader (Molecular Devices, USA).

#### Peptides Activity on Intracellular Amastigotes

Macrophages obtained from the peritoneal cavity of BALB/c mice were plated at 5 × 10^5^ cells/well in a 24-well plate and incubated overnight at 37 ^o^C and 5% CO_2_ for adhesion. Infection was performed with 10 parasites per macrophage using *L. (L.) amazonensis* and *L. (V.) braziliensis* at the stationary phase. The parasites were allowed to enter macrophages for 3 h, after which the wells were washed to remove external parasites and the peptides were added at a concentration of 50 μM. After 24 h of treatment, the cells were washed, fixed and stained. Analysis was performed by counting the average number of amastigotes in 200 macrophages under light microscopy in triplicate.

### Mimetic Membrane Assays

#### Reagents

Avanti Polar Lipids Inc. (Alabaster, AL, USA) supplied the phospholipids 1-palmitoyl-2-oleoyl-glycero-3-phosphocoline (POPC), 1-palmitoyl-2-oleoyl-*sn*-glycero-3-phospho­(1′-rac-glycerol) (POPG), 1-palmitoyl-2-oleoyl-*sn*-glycero-3-phospho-l-serine (POPS), 1-palmitoyl-2-oleoyl-*sn*-glycero-3-phosphoethanolamine (POPE), lysophosphatidylcholine (L), sphingomyelin (SPM), cholesterol (Col), and ergosterol (Erg). All other reagents were supplied by Sigma and/or were >95% pure by HPLC.

#### Large Unilamellar Vesicle (LUV) Preparations

According to previously described procedures, the hydration of lipid films of the desired lipid mixture with appropriate buffers at 10 ^o^C above the phase transition temperature, followed by extrusion through polycarbonate membranes, yielded suspensions of LUVs (∼130 nm in diameter).^39^ The following lipid compositions were prepared: PE/PS/Erg (47:28:25) - mimicking promastigote membranes,^15^ PC/SPM/Col (42:35:23) - mimicking erythrocyte membrane,^17,18^ PC/PE/PS/L (37:32:18:13) - mimicking macrophage-infected membranes,^16^ and PC/PS (71:29), PE/PS (71:29), PC/PE/PS (47:35:18) as references. For experiments of zeta potential, size and circular dichroism (CD), lipid films were hydrated with citrate/phosphate buffer, containing 150 mM NaF at pH 5.5; and for the translocation assays, the citrate/phosphate buffer at pH 5.5 containing 150 mM NaCl. For the leakage experiments, the vesicle suspension was initially hydrated with a solution of 12.5 mM ANTS and 45 mM DPX dissolved in citrate/phosphate buffer containing 50 mM NaCl at pH 5.5. After extrusion, the vesicle suspension was subjected to gel filtration (PD-10 column, Sephadex G-25, GE Healthcare) to separate unencapsulated ANTS/DPX. The column was eluted with the citrate/phosphate buffer containing 150 mM NaCl at pH 5.5.

#### Circular Dichroism (CD) Measurements

CD spectra were collected for 20 μM peptide solutions in buffer at pH 5.5, in 2,2,2- trifluoroethanol (40% TFE in water) and in sodium dodecyl sulfate micellar solution (8 mM SDS), as well as in the presence of 100, 200, and 300 μM LUVs suspensions at the desired lipid compositions. Spectra were recorded from 260 to 190 nm using a Jasco-815 spectropolarimeter (JASCO International Co Ltd., Tokyo, Japan) at 25 °C, with a 0.5 cm path length cuvette and 10 accumulations. The observed ellipticity (θ (mdeg)) was corrected for dilution and subtracted from the respective baseline. The data were then converted to mean residue ellipticity ([Θ], deg cm^2^/dmol) according to (eq 1): [Θ] = 100 *l*.*c*.(*n* – 1), where *l* is the path length in cm, *c* is the peptide concentration in millimolar, and *n* is the number of peptide bonds. The Dichroweb website (Birkbeck, London College, London, UK)^37^ was used to assign the contributions of the peptide secondary structures employing the CDSSTR calculation algorithm.^50^

#### Zeta Potential and Partition Coefficient (K_P_) Determination

Zeta potentials (ζ) of pure LUVs and in the presence of increased peptide concentration were determined using Zetasizer Nano ZS (Malvern Instruments, Malvern, Worcestershire, UK). The zeta potential is calculated through the electrophoretic mobility (μ), using the Smoluchowski equation (eq 2): $\mu =\frac{\varepsilon_{r}\varepsilon_{0}}{\eta }\zeta f\left(\kappa R\right)$ , where ε_*r*_ and ε_0_ are the dielectric constant of water (78.5) and the free space permittivity, respectively, η is the water viscosity (0.8873 cP), *R* is the average vesicles radius, κ is the inverse of the Debye length, *f*(κ*R*) is the Henry function.^51^ Each sample was equilibrated for 15 min at 25 °C and transferred to DTS1070 (Malvern Instruments) cuvettes for zeta potential measurement. Considering that each peptide charge neutralizes one lipid charge, the fraction of lipid neutralized by peptide (X_L_) can be determined through the evaluation of zeta values according to eq 3: $\frac{\zeta }{\zeta_{0}}=1+\frac{X_{L}z_{pep}}{f_{PL}\left[L\right]z_{lip}}.\left[P\right]$ , where z_pep_ and z_lip_ are, respectively, the net charges of the peptides and lipids; ζ and ζ_0_ are, respectively, the zeta potential values at each peptide concentration and in the absence of peptide;, [L] and [P] are the concentrations of lipid and peptide, respectively, and f_PL_ the fraction of anionic phospholipids in the vesicles. From this relationship, the partition coefficient (K_P_) can be obtained from the dependence of X_L_ on lipid concentration (eq 4) $X_{L}=\frac{K_{P}\gamma_{L}\left[L\right]}{1+K_{P}\gamma_{L}\left[L\right]}$ ^38^ where γ_L_ is the lipid molar volume.^52,53^

#### Dye Leakage

ANTS/DPX leakage experiments were monitored by fluorescence emission spectra using an ISS PC1 spectrofluorometer (Urbana, Champaign, IL, USA), at 25 °C. Aliquots of the vesicle suspension were added, under stirring, to the 1 cm path length cuvette containing the peptide solution in citrate/phosphate/NaCl buffer at pH 5.5. The total contact time for the experiments was 15 min. Fluorescence emission at 520 nm (Ex. 360 nm) was measured over time to determine the probe fluorescent intensity released using eq 5: %Leakage = 100 × (*F*–*F*_0_)/(*F*_100_–*F*_0_) where *F* is the fluorescence intensity measured (arbitrary units), *F*_0_ and *F*_100_ are the fluorescence intensities in the absence of peptides and after complete release of vesicle contents, respectively. *F*_100_ was obtained by adding 12 μL of a 10% Triton X-100 solution, with corrections applied for the dilution factor. The experiments were performed in duplicate.

#### Dynamic Light Scattering (DLS) for LUVs Size Measurement

The vesicles diameters (*d*) were determined by light scattering, through the use of a laser light source He–Ne (633 nm), operating at a scattering angle of 173° on the same equipment used for zeta potential measurements, considering different concentrations of the peptides. Specific disposable cells for size evaluation (Sarstedt ref. 67.754, Nümbrecht, Germany) were used. *d* was calculated from diffusion coefficient (D) using the Stokes–Einstein relation (eq 6): $d=\frac{k_{B}T}{3\pi \eta D}$ , where *k*_B_ is the Boltzmann constant and *T* is the temperature.

#### Diphenylhexatriene (DPH) Fluorescence Anisotropy

DPH was incorporated at low proportion (∼0.001) in the preparation of lipid films. Buffered solutions of peptides at increasing concentrations were prepared, to which 50 μM vesicles were added.^54^ Samples were prepared directly into disposable cuvettes (Sarstedt Ref. 67755, Nümbrecht, Germany) and allowed to stand for 4 h at room temperature and protected from light. DPH emission fluorescence spectra were collected using a ISS PC1 spectrofluorometer (Urbana Champaign, Il, USa), equipped with Glan-Thompson polarizers, at 25 °C, with excitation and emission wavelengths set at 360 and 450 nm and bandwidth at 2 and 1 nm, respectively. Fluorescence anisotropy is given by the equation: *r* = (*F*_//_ – *F*_⊥_) (*F*_//_ + 2*F*_⊥_) where *F*_//_ is the fluorescence intensity parallel to the excitation plane and *F*_⊥_ is the perpendicular component to same plan.^42^

#### Translocation Assay

Peptide translocation across lipid bilayers was assessed based on the intrinsic fluorescence of tryptophan residues using negatively charged lipid vesicles mimicking macrophage-infected membranes, following a two-stage protocol comprising release and dilution experiments.^40,44^ In the release stage, LUVs composed of PC/PE/PS/L (5 mM total lipid concentration) were prepared by hydrating a dry lipid film with a buffered peptide solution (25 μM), followed by extrusion. The resulting suspension was diluted tenfold and successive aliquots (10 × 15 μL) were added to a cuvette containing 1.5 mL of buffer. Subsequently, additional aliquots of the initial stock suspension (10 × 15 μL and 10 × 75 μL) were injected. After each addition, a spectrum was recorded. This procedure allows monitoring the release of the peptide molecules associated with the inner and outer leaflets of the membranes. The extent of this release reflects the degree of translocation, with complete desorption expected only for membrane-permeant peptides. In the dilution stage, LUVs (5 mM PC/PE:PS/L) were first prepared and then mixed with a 25 μM peptide solution, homogenized, and diluted tenfold. The same titration protocol as described for the release stage was then applied. Under these strong dilution conditions, peptides are expected to desorb from the membrane, regardless of their ability to translocate providing a reference for complete peptide release. Fluorescence emission spectra were acquired using an ISS PC1 spectrofluorimeter (Urbana, Champaign, IL, USA) equipped with Glan–Thompson polarizers (excitation at 90° and emission at 0°), at 25 °C, over the range of 300–500 nm, with excitation at 280 nm and bandwidth of 2 nm. Measurements were performed using a 1 cm path length quartz cuvette (3ES10, Fireflysci, Brooklyn, NY). Fluorescence emission spectra were normalized in relation to peptide concentration. The fraction of membrane bound peptide (υ) is calculated by $\upsilon =\frac{\Sigma \left(F\left(\lambda \right)-F_{aq}\left(\lambda \right)\right)\left(F_{b}\left(\lambda \right)-F_{aq}\left(\lambda \right)\right)}{\Sigma {\left(F_{b}\left(\lambda \right)-F_{aq}\lambda \right)}^{2}}$ (eq 7), where *F*(λ) is the fluorescence intensity at the wavelength (λ), *F*_aq_(λ) is the corresponding value of free peptide in aqueous solution measured in the absence of vesicles; *F*_b_(λ) is the corresponding value of completely membrane-bound peptide, measured in the presence of excess lipid. Comparing plots of the release and dilution stages of the experiment, respective υ values will indicate: if υ of both experiments superimpose means that the peptide could equilibrate across the vesicle membranes; if υ values of the dilution experiment are lower than those of the release stage, the peptide molecules do not equilibrate across the vesicle membranes during the experiment time course. Considering that the release and dilution stages just differ by the way stock solutions were prepared, discrepancies between υ values of these experiments can be explained only by the transbilayer distribution of the peptide.^40^

## Supplementary Material



## Notes

Viability/survival of promastigotes; plasma membrane permeabilization induced by peptide DecP-9 in *Leishmania (L.) infantum* promastigotes; CD spectra of peptides acquired in buffer at pH 5.5, TFE (40%), and SDS (8 mM); zeta potential isotherms; and normalization of zeta potential data to determine the partitioning (K_*P*_) of peptides (PDF)
