# Supplementary material for: Assessing Designed Model Membrane-Targeting Peptides Found as Potential Molecular Scaffolds against Cutaneous Leishmaniasis
Source: ACS Omega. 2026 Sep 16;11(38):57046–60. doi: 10.1021/acsomega.6c04342 (PMC13629047; doi:10.1021/acsomega.6c04342)
Supplement: Supplementary file 1 [file ao6c04342_si_001.pdf]

Assessing Designed Model Membrane-Targeting Peptides Found as Potential Molecular Scaffolds against Cutaneous Leishmaniasis

Viviane Ap. C. Santana[1], Danúbia B. Martins[2], Vanessa Albuquerque[3], Anna Karoline Aguiar Fleuri Balbi[5], José Eduardo Ferraz Souza[2], Andre Gustavo Tempone[3], Marcelo Dias-Baruffi[4], Thalita Riul[4,5], Dayane S. Alvares[2], Marcia Perez dos Santos Cabrera[1,2*].*

[1]Departamento de Química e Ciências Ambientais e [2]Departamento de Física, Universidade Estadual Paulista, 15054-000 São José do Rio Preto, SP, Brasil

[3]Laboratório de Fisiopatologia, Instituto Butantan, 05503-900 São Paulo, SP, Brasil

[4]Departamento de Análises Clínicas Toxicológicas e Bromatológicas - Universidade de São Paulo, 14040-903 Ribeirão Preto, SP, Brasil

[5]Faculdade de Ciências Farmacêuticas, Alimentos e Nutrição - Universidade Federal do Mato Grosso do Sul, 79070-900 Campo Grande, MS, Brasil

*Corresponding authors

E-mail: cabrera.marcia@gmail.com (MPDSC); dayane.alvares@unesp.br (DSA)

SUPPLEMENTARY INFORMATION

Contents

Figure SI1. Viability/survival of promastigotes.

Figure SI2. Plasma membrane permeabilization induced by peptide DecP-9 in *Leishmania (L.) infantum* promastigotes.

Figure SI3. CD spectra of peptides acquired in buffer at pH 5.5, TFE (40%), and SDS (8 mM).

Figure SI4. Zeta potential isotherms.

Figure SI5. Normalization of zeta potential data to determine the partitioning (K_P) of peptides.

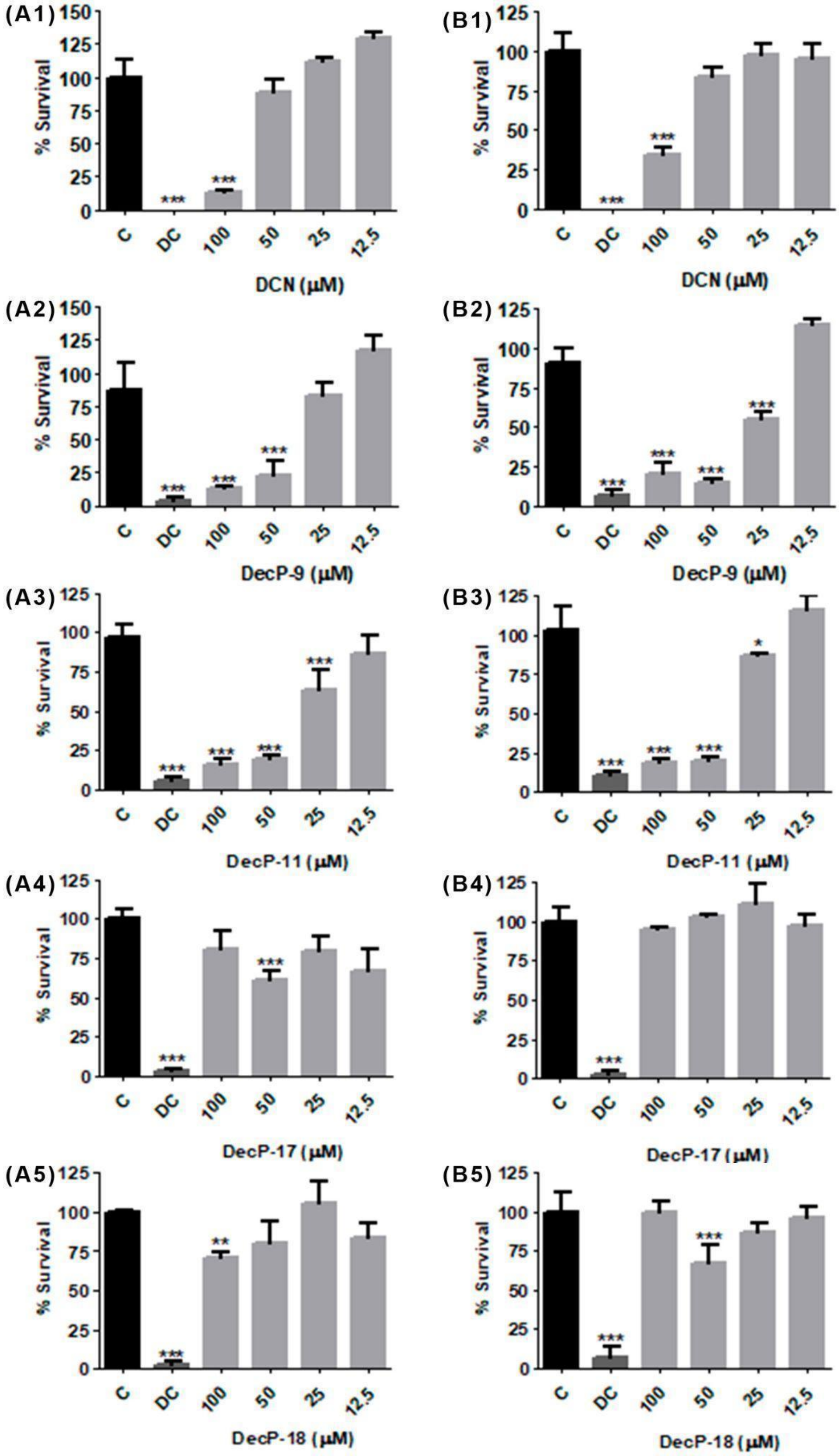

Figure SI1. Viability/survival of promastigotes. *Leishmania sp.* promastigotes susceptibility to different peptides. (A1 - A5) *L. amazonensis*; (B1 - B5) *L. braziliensis* promastigotes treated with different peptides; after 24 h cell viability was assessed by MTT assay. C, negative control; DC, death control treated with 10 µM amphotericin B. ** and *** represent p<0,01 and p<0,001, respectively (One-way ANOVA with Tukey's post hoc test).

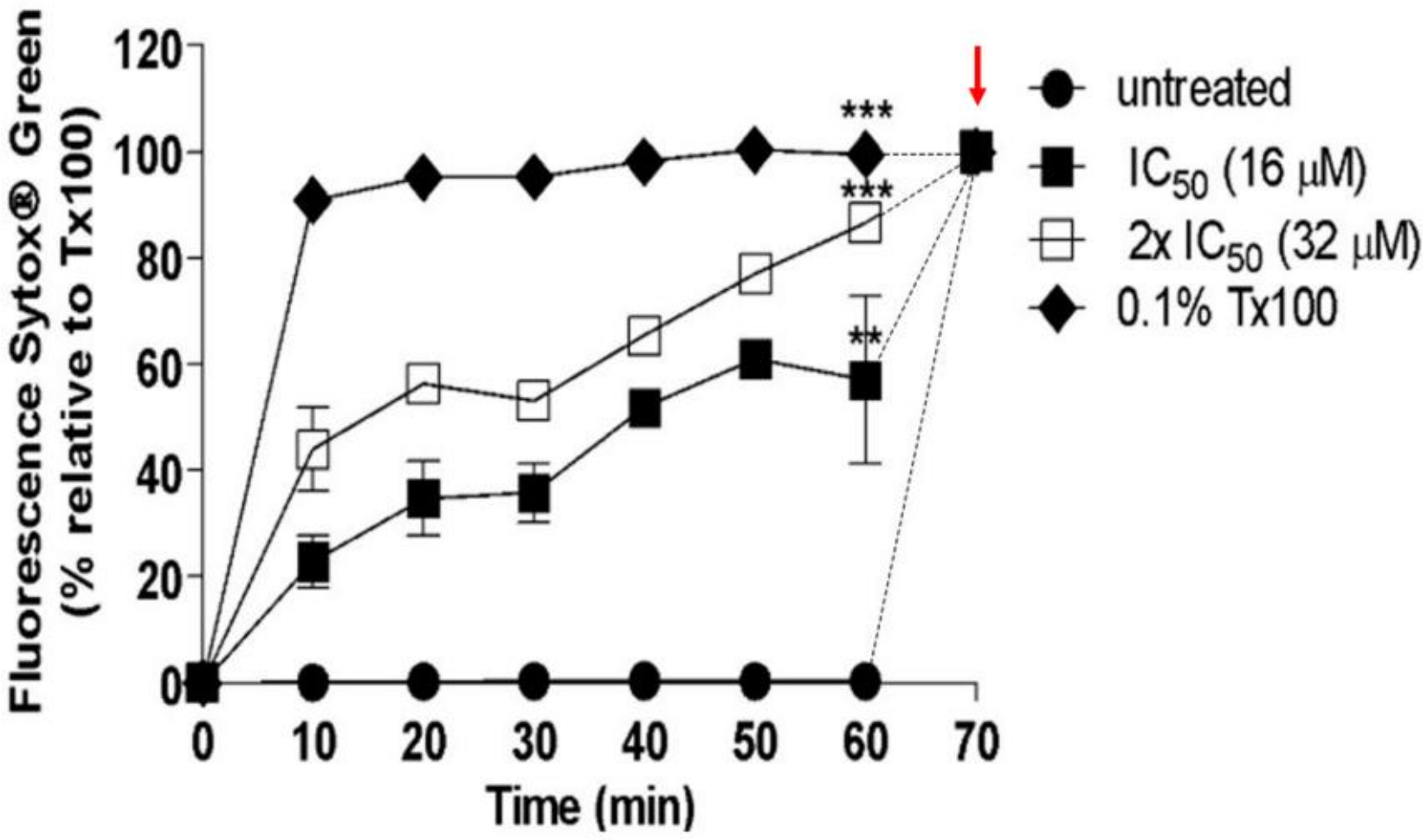

Figure SI2. Plasma membrane permeabilization induced by peptide DecP-9 in *Leishmania (L.) infantum* promastigotes. The entrance of Sytox Green dye was monitored fluorometrically during 1 h incubation in the presence of peptide DecP-9. Treated promastigotes with 0.1% Triton X-100 and untreated promastigotes were used as positive and negative controls, respectively. The red arrow indicates the addition of Triton X-100 to each experimental condition. A representative assay is shown. **$p<0.01$; ***$p<0.001$.

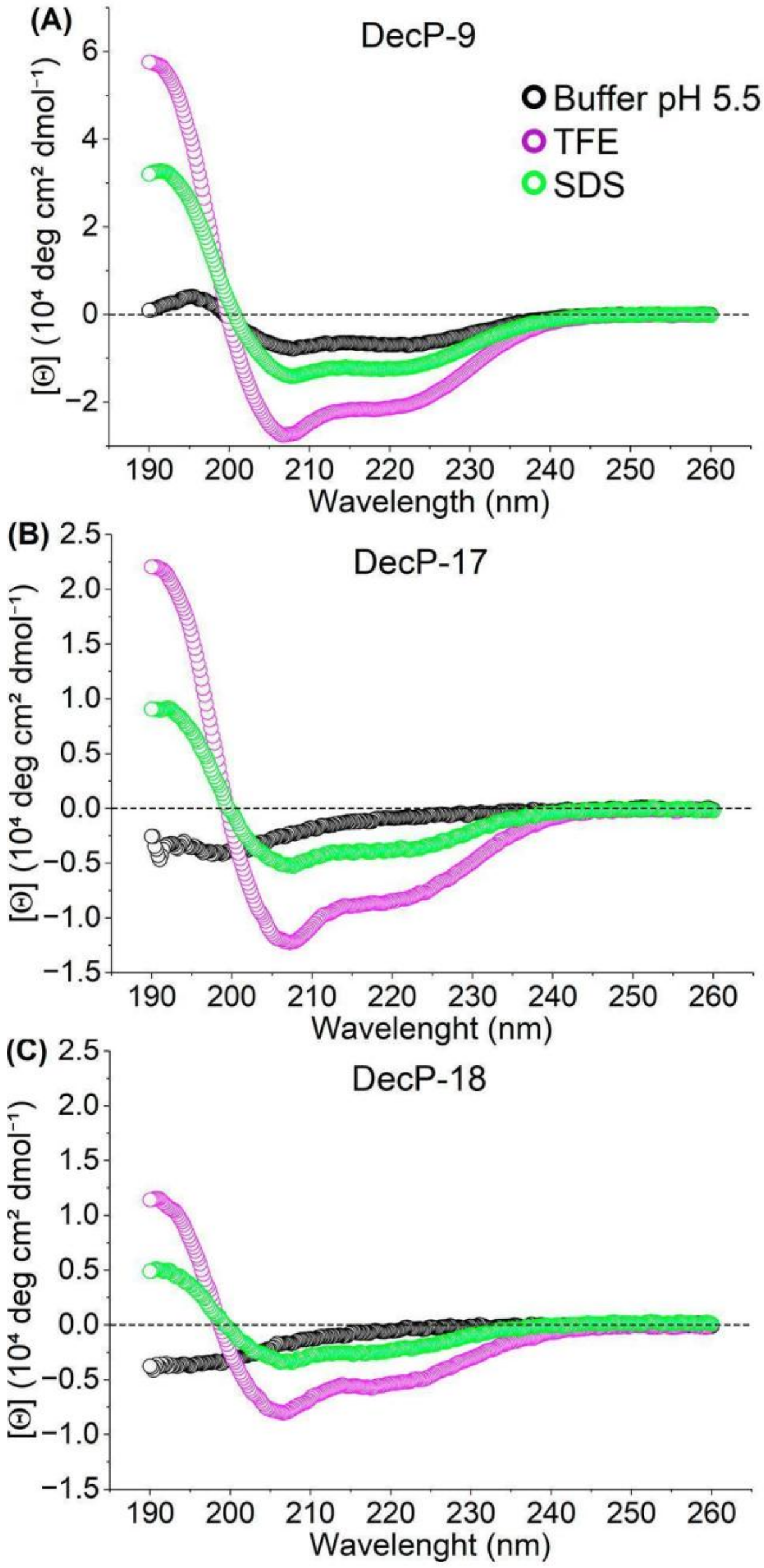

Figure SI3. CD spectra of 20 μM peptides obtained at 25 °C in buffer (pH 5.5), TFE (40%), and SDS (8 mM). (A) DecP-9, (B) DecP-17, and (C) DecP-18.

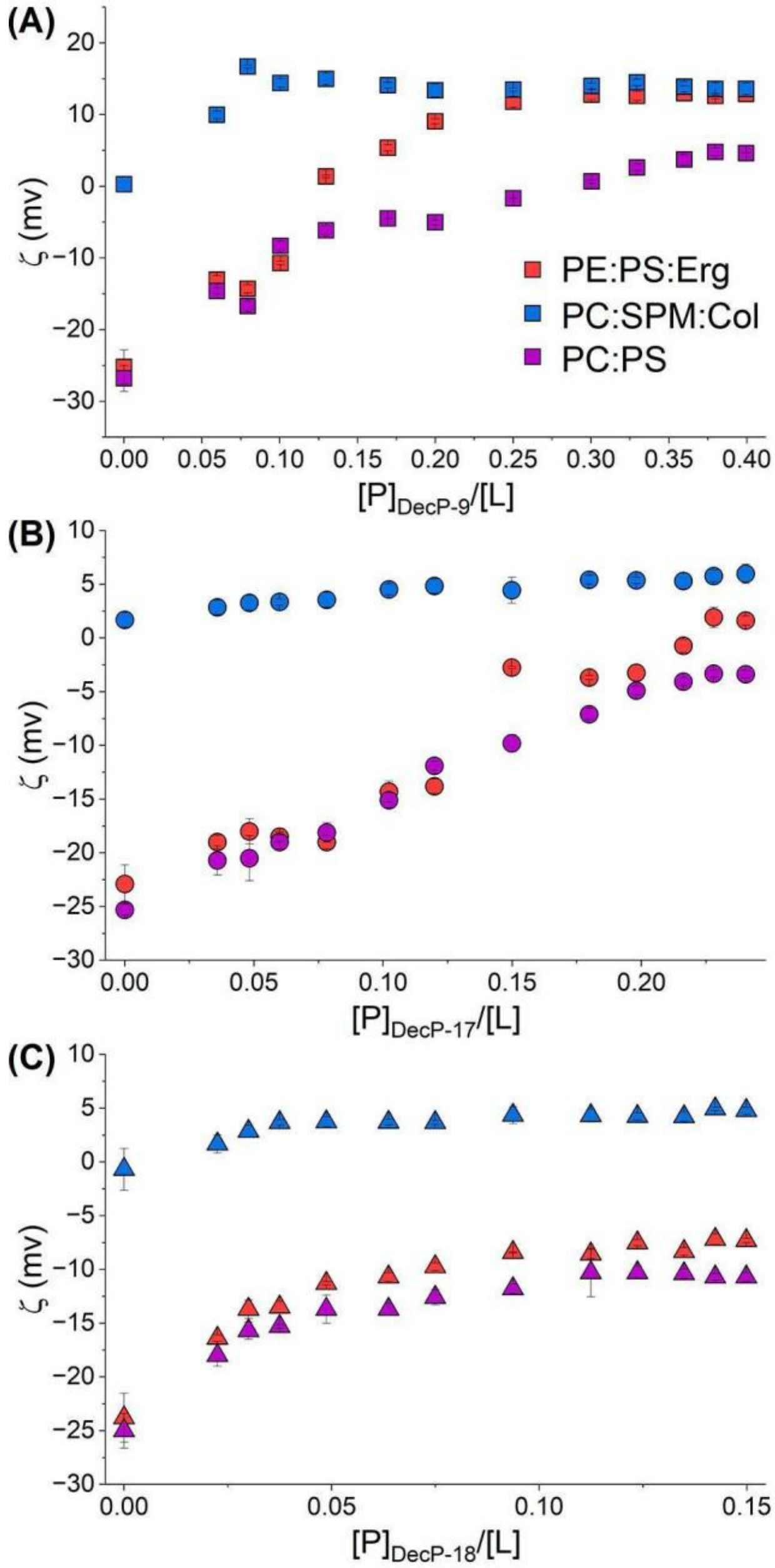

Figure SI4. Zeta potential isotherms for LUVs in the absence and in the presence of increasing peptide concentrations (25 °C): (A) DecP-9, (B) DecP-17, and (C) DecP-18. PE:PS:Erg (red), mimicking promastigote membranes; PC:SPM:Col (blue), mimicking mammalian cell membranes; and PC:PS (purple), as a reference of high negative charge content.

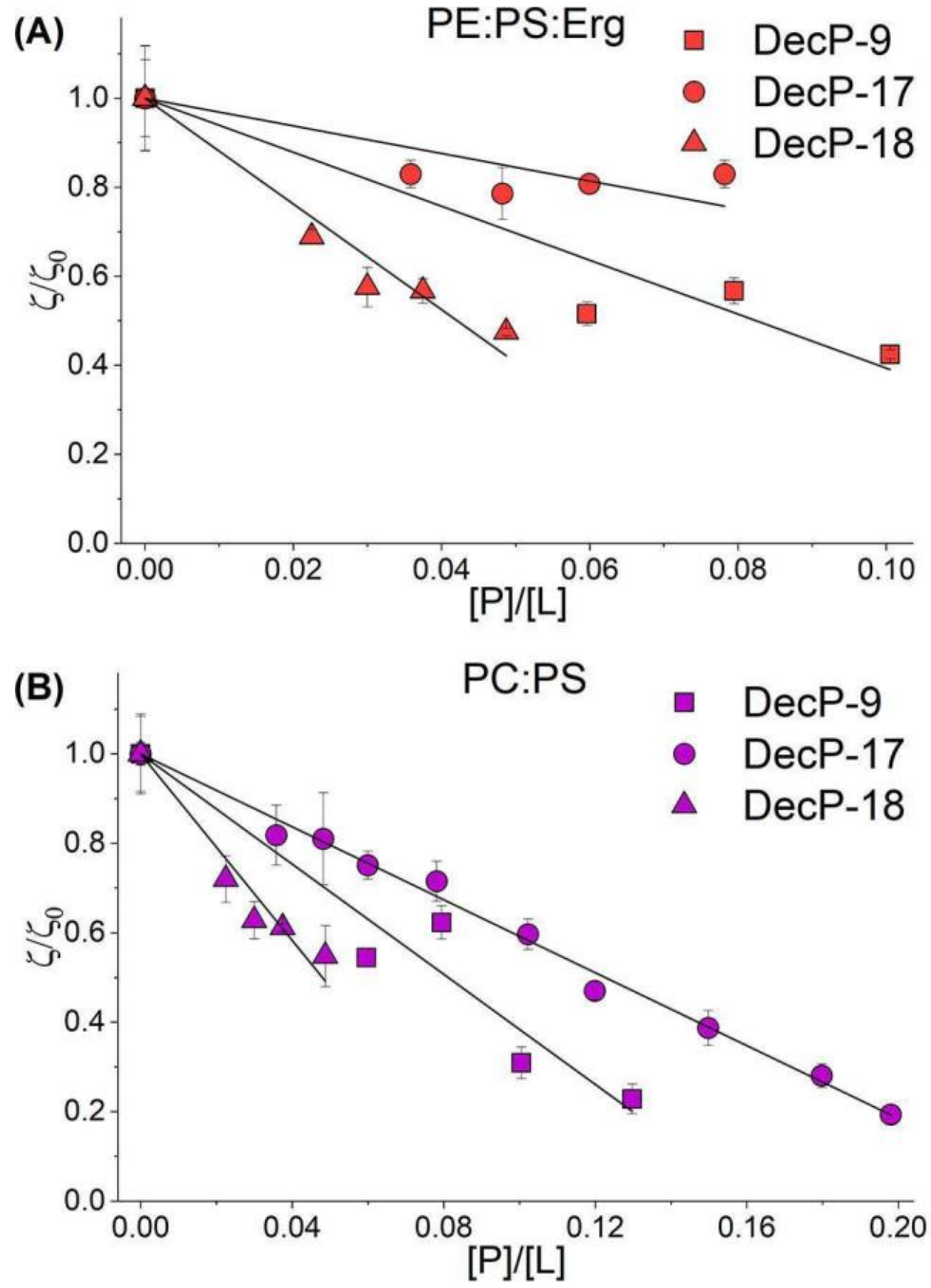

Figure SI5. Normalization of zeta potential values as a function of peptide/lipid molar ratio considering the LUVs composed of: (A) PE:PS:Erg, and (B) PC:PS. The continuous lines represent the fitting according to Eq. 3 to determine the partition coefficients (K_P).
